# Supplementary material for: Predictive Validity of Motor Fitness and Flexibility Tests in Adults and Older Adults: A Systematic Review
Source: J Clin Med. 2022 Jan 10;11(2):328. doi: 10.3390/jcm11020328 (PMC8779466; doi:10.3390/jcm11020328)
Supplement: Supplementary file 1 [file jcm-11-00328-s001.zip › jcm-1466181-supplementary/SupplementaryFigure1.pdf]

Supplementary Figure S1. Number of studies reporting predictive validity or lack of predictive validity of the included motor fitness and flexibility tests for different health outcomes.

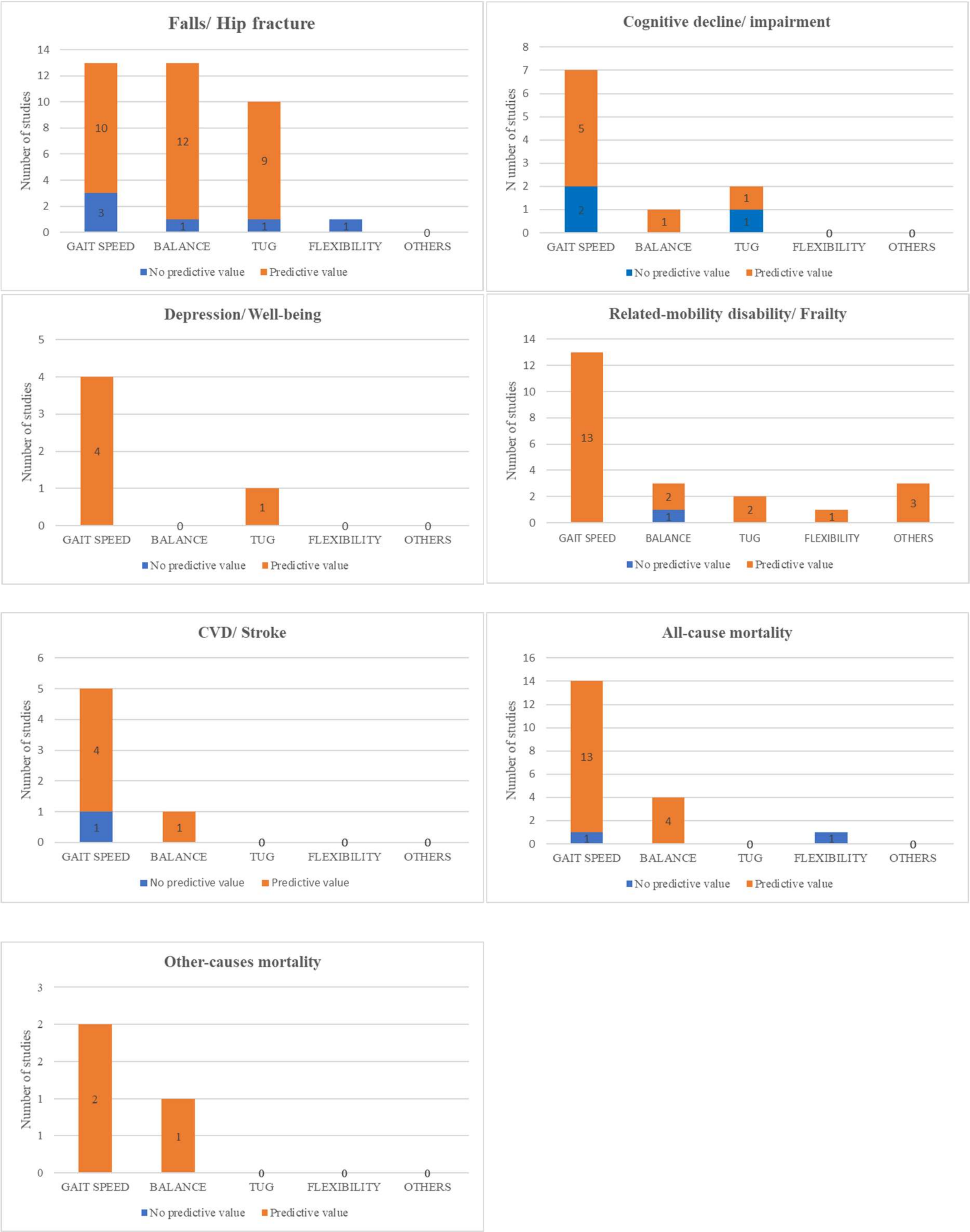

CVD, Cardiovascular Disease; TUG, Timed Up&Go test; “Other” includes: maximum step length, speed of movement and stair mounting test.
