# Supplementary material for: Predictive Validity of Motor Fitness and Flexibility Tests in Adults and Older Adults: A Systematic Review
Source: J Clin Med. 2022 Jan 10;11(2):328. doi: 10.3390/jcm11020328 (PMC8779466; doi:10.3390/jcm11020328)
Supplement: Supplementary file 1 [file jcm-11-00328-s001.zip › jcm-1466181-supplementary/SupplementaryFigure2.pdf]

Supplementary Figure S2. Cut points of gait speed at usual pace and risk of adverse outcomes found in the selected studies and systematic reviews/meta-analysis.

<1.3m/s	Hip fracture [1]; Depressive symptoms [2]; CVD risk [3]; All-cause mortality [3,4]
<1.2m/s	Mobility disability [5]; IADL [6]; All-cause mortality [6]
<1.1m/s	Mobility disability [7,8]; IADL [9,10]; CVD risk [11]; All-cause mortality [11–13]; Other-causes mortality [11]
<1.0m/s	Mobility disability [14–16]; IADL [16,17]; Cognitive decline/impairment/Alzheimer [16, 18]; CVD risk [4]; Hospitalization/Institutionalization [15,16]; All-cause mortality [16]; Other-causes mortality [19]
<0.9m/s	IADL [9]; CVD risk [11]; All-cause mortality [11]; Other-causes mortality [11]
<0.8m/s	Falls [20]; IADL [21]; Frailty [22]; Cognitive decline/Dementia [23, 24]; Depressive symptoms [25]; CVD risk [21]; Hospitalization [26]; All-cause mortality [22,23,26,27]
<0.7m/s	Falls [16,28,29]; Cognitive decline [30]; All-cause mortality [28]
<0.6m/s	Falls [31,32]; IADL [15]
<0.5m/s	Depressive symptoms [33]
<0.4m/s	Hip fracture [34]

CVD, Cardiovascular disease risk; IADL, Instrumental Disability in Daily Activities.

REFERENCES

1. Wihlborg A, Englund M, Akesson K, Gerdhem P. Fracture predictive ability of physical performance tests and history of falls in elderly women: a 10-year prospective study. Osteoporosis International. 2015;26(8):2101-9.
2. Briggs R, Carey D, Claffey P, McNicholas T, Donoghue O, Kennelly SP, et al. Do Differences in Spatiotemporal Gait Parameters Predict the Risk of Developing Depression in Later Life? Journal of the American Geriatrics Society. 2019;67(5):1050-6.
3. Georgiopoulou VV, Kalogeropoulos AP, Chowdhury R, Binongo JNG, Bibbins-Domingo K, Rodondi N, et al. Exercise Capacity, Heart Failure Risk, and Mortality in Older Adults: The Health ABC Study. American Journal of Preventive Medicine. 2016;52:144-53.
4. Niiranen TJ, Enserro DM, Larson MG, Vasan RS. Multisystem Trajectories Over the Adult Life Course and Relations to Cardiovascular Disease and Death. Journals of Gerontology Series a-Biological Sciences and Medical Sciences. 2019;74(11):1778-85.
5. Deshpande N, Metter EJ, Guralnik J, Bandinelli S, Ferrucci L. Predicting 3-Year Incident Mobility Disability in Middle-Aged and Older Adults Using Physical Performance Tests. Archives of Physical Medicine and Rehabilitation. 2013;94:994-7.
6. Abe T, Kitamura A, Taniguchi Y, Amano H, Seino S, Yokoyama Y, et al. Pathway from gait speed to incidence of disability and mortality in older adults: A mediating role of physical activity. Maturitas. 2019;123:32-6.
7. Doi T, Nakakubo S, Tsutsumimoto K, Kim MJ, Kurita S, Ishii H, et al. Spatio-temporal gait variables predicted incident disability. Journal of Neuroengineering and Rehabilitation. 2020;17(1).
8. Rosso AL, Metti AL, Faulkner K, Brach JS, Studenski SA, Redfern M, et al. Associations of Usual Pace and Complex Task Gait Speeds With Incident Mobility Disability. Journal of the American Geriatrics Society. 2019;67(10):2072-6.
9. Makizako H, Shimada H, Doi T, Tsutsumimoto K, Lee S, Hotta R, et al. Cognitive Functioning and Walking Speed in Older Adults as Predictors of Limitations in Self-Reported Instrumental Activity of Daily Living: Prospective Findings from the Obu Study of Health Promotion for the Elderly. International Journal of Environmental Research and Public Health. 2015;12:3002-13.
10. Adachi T, Kamiya K, Kono Y, Iwatsu K, Shimizu Y, Honda I, et al. Estimation of reduced walking speed using simple measurements of physical and psychophysiological function in community-dwelling elderly people: a cross-sectional and longitudinal study. Aging Clinical and Experimental Research. 2019;31:59-66.
11. Nofuji Y, Shinkai S, Taniguchi Y, Amano H, Nishi M, Murayama H, et al. Associations of Walking Speed, Grip Strength, and Standing Balance With Total and Cause-Specific Mortality in a General Population of Japanese Elders. Journal of the American Medical Directors Association. 2016;17.
12. Elbaz A, Sabia S, Brunner E, Shipley M, Marmot M, Kivimaki M, et al. Association of walking speed in late midlife with mortality: results from the Whitehall II cohort study. Age. 2013;35:943-52.

13. Idland G, Pettersen R, Avlund K, Bergland A. Physical performance as long-term predictor of onset of activities of daily living (ADL) disability: A 9-year longitudinal study among community-dwelling older women. Archives of Gerontology and Geriatrics. 2013;56:501-6.
14. Brach JS, Wert D, VanSwearingen JM, Newman AB, Studenski SA. Use of Stance Time Variability for Predicting Mobility Disability in Community-Dwelling Older Persons: A Prospective Study. Journal of Geriatric Physical Therapy. 2012;35:112-7.
15. Cavanaugh EJ, Richardson J, McCallum CA, Wilhelm M. The Predictive Validity of Physical Performance Measures in Determining Markers of Preclinical Disability in Community-Dwelling Middle-Aged and Older Adults: A Systematic Review. Physical Therapy. 2018;98(12):1010-21.
16. van Kan GA, Rolland Y, Andrieu S, Bauer J, Beauchet O, Bonnefoy M, et al. GAIT SPEED AT USUAL PACE AS A PREDICTOR OF ADVERSE OUTCOMES IN COMMUNITY-DWELLING OLDER PEOPLE AN INTERNATIONAL ACADEMY ON NUTRITION AND AGING (IANA) TASK FORCE. Journal of Nutrition Health & Aging. 2009;13(10):881-9.
17. Wang DXM, Yao J, Zirek Y, Reijnierse EM, Maier AB. Muscle mass, strength, and physical performance predicting activities of daily living: a meta-analysis. Journal of Cachexia Sarcopenia and Muscle. 2020;11(1):3-25.
18. Tian Q, Resnick SM, Studenski SA, Ferrucci L. Lap Time Variability From a 400-m Walk Is Associated With Future Mild Cognitive Impairment and Alzheimer's Disease. Journal of the American Medical Directors Association. 2019;20(12):1535-+.
19. Looker AC. Dysmobility syndrome and mortality risk in US men and women age 50 years and older. Osteoporosis International. 2015;26:93-102.
20. Callisaya ML, Ayers E, Barzilai N, Ferrucci L, Guralnik JM, Lipton RB, et al. Motoric Cognitive Risk Syndrome and Falls Risk: A Multi-Center Study. Journal of Alzheimers Disease. 2016;53:1043-52.
21. Heiland EG, Welmer AK, Wang R, Santoni G, Fratiglioni L, Qiu CX. Cardiovascular Risk Factors and the Risk of Disability in Older Adults: Variation by Age and Functional Status. Journal of the American Medical Directors Association. 2018;20:208-+.
22. Jung HW, Jang IY, Lee CK, Yu SS, Hwang JK, Jeon C, et al. Usual gait speed is associated with frailty status, institutionalization, and mortality in community-dwelling rural older adults: a longitudinal analysis of the Aging Study of Pyeongchang Rural Area. Clinical Interventions in Aging. 2018;13:1079-89.
23. Hoogendijk EO, Rijnhart JJM, Skoog J, Robitaille A, van den Hout A, Ferrucci L, et al. Gait speed as predictor of transition into cognitive impairment: Findings from three longitudinal studies on aging. Experimental Gerontology. 2020;129.
24. Grande G, Triolo F, Nuara A, Welmer AK, Fratiglioni L, Vetrano DL. Measuring gait speed to better identify prodromal dementia. Experimental Gerontology. 2019;124.

25. Veronese N, Stubbs B, Trevisan C, Bolzetta F, De Rui M, Solmi M, et al. Poor Physical Performance Predicts Future Onset of Depression in Elderly People: Progetto Veneto Anziani Longitudinal Study. Physical Therapy. 2017;97:659-68.
26. Zucchelli A, Vetrano DL, Grande G, Calderon-Larranaga A, Fratiglioni L, Marengoni A, et al. Comparing the prognostic value of geriatric health indicators: a population-based study. Bmc Medicine. 2019;17(1).
27. Blain H, Carriere I, Sourial N, Berard C, Favier F, Colvez A, et al. Balance and walking speed predict subsequent 8-year mortality independently of current and intermediate events in well-functioning women aged 75 years and older. Journal of Nutrition Health & Aging. 2010;14:595-600.
28. Sanders JB, Bremmer MA, Comijs HC, van de Ven PM, Deeg DJH, Beekman ATF. Gait Speed and Processing Speed as Clinical Markers for Geriatric Health Outcomes. American Journal of Geriatric Psychiatry. 2016;25:374-85.
29. Luukinen H, Koski K, Laippala P, Kivela SL. PREDICTORS FOR RECURRENT FALLS AMONG THE HOME-DWELLING ELDERLY. Scandinavian Journal of Primary Health Care. 1995;13(4):294-9.
30. Stijntjes M, Aartsen MJ, Taekema DG, Gussekloo J, Huisman M, Meskers CGM, et al. Temporal Relationship Between Cognitive and Physical Performance in Middle-Aged to Oldest Old People. Journals of Gerontology Series a-Biological Sciences and Medical Sciences. 2017;72:662-8.
31. Quach L, Galica AM, Jones RN, Procter-Gray E, Manor B, Hannan MT, et al. The Nonlinear Relationship Between Gait Speed and Falls: The Maintenance of Balance, Independent Living, Intellect, and Zest in the Elderly of Boston Study. Journal of the American Geriatrics Society. 2011;59:1069-73.
32. Doi T, Hirata S, Ono R, Tsutsumimoto K, Misu S, Ando H. The harmonic ratio of trunk acceleration predicts falling among older people: results of a 1-year prospective study. Journal of Neuroengineering and Rehabilitation. 2013;10.
33. Sanders JB, Bremmer MA, Deeg DJH, Beekman ATF. Do Depressive Symptoms and Gait Speed Impairment Predict Each Other's Incidence? A 16-Year Prospective Study in the Community. Journal of the American Geriatrics Society. 2012;60:1673-80.
34. Kauppi M, Stenholm S, Impivaara O, Maki J, Heliovaara M, Jula A. Fall-related risk factors and heel quantitative ultrasound in the assessment of hip fracture risk: a 10-year follow-up of a nationally representative adult population sample. Osteoporosis International. 2014;25:1685-95.
