# Supplementary material for: Predictive Validity of Motor Fitness and Flexibility Tests in Adults and Older Adults: A Systematic Review
Source: J Clin Med. 2022 Jan 10;11(2):328. doi: 10.3390/jcm11020328 (PMC8779466; doi:10.3390/jcm11020328)
Supplement: Supplementary file 1 [file jcm-11-00328-s001.zip › jcm-1466181-supplementary/SupplementaryFigure3.pdf]

Supplementary Figure S3. Cut points of timed up&go test and risk of adverse outcomes found in the selected studies and systematic reviews/meta-analysis.

>7s	Falls [1]
>8s	Falls [2,3]
>10s	Fear of falling [4]
>11s	Falls [5]; Functional autonomy decline [6]
>12s	Falls [7]; IADL [8]
>13s	Falls [9]
>16s	Falls [10]; Frailty [11]
>20s	Well-being decline [12]
>21s	Falls [13]

IADL, Instrumental Disability in Daily Activities.

REFERENCES

1. Asai T, Oshima K, Fukumoto Y, Yonezawa Y, Matsuo A, Misu S. Does dual-tasking provide additional value in timed "up and go" test for predicting the occurrence of falls? A longitudinal observation study by age group (young-older or old-older adults). Aging Clinical and Experimental Research. 2020.
2. Abu Samah Z, Singh DKA, Murukesu RR, Shahar S, Nordin NAM, Omar MA, et al. Discriminative and Predictive Ability of Physical Performance Measures in Identifying Fall Risk among Older Adults. Sains Malaysiana. 2018;47:2769-76.
3. Nitz JC, Stock L, Khan A. Health-related predictors of falls and fractures in women over 40. Osteoporosis international : a journal established as result of cooperation between the European Foundation for Osteoporosis and the National Osteoporosis Foundation of the USA. 2013;24:613-21.
4. Austin N, Devine A, Dick I, Prince R, Bruce D. Fear of falling in older women: a longitudinal study of incidence, persistence, and predictors. Journal of the American Geriatrics Society. 2007;55(10):1598-603.
5. Kwan MM, Lin SI, Close JC, Lord SR. Depressive symptoms in addition to visual impairment, reduced strength and poor balance predict falls in older Taiwanese people. Age and ageing. 2012;41(5):606-12.
6. Breton E, Beloin F, Fortin C, Martin A, Ouellet ME, Payette H, et al. Gender-specific associations between functional autonomy and physical capacities in independent older adults: Results from the NuAge study. Archives of Gerontology and Geriatrics. 2014;58:56-62.
7. Clemson L, Kendig H, Mackenzie L, Browning C. Predictors of Injurious Falls and Fear of Falling Differ: An 11-Year Longitudinal Study of Incident Events in Older People. Journal of Aging and Health. 2015;27:239-56.
8. Wang DXM, Yao J, Zirek Y, Reijnierse EM, Maier AB. Muscle mass, strength, and physical performance predicting activities of daily living: a meta-analysis. Journal of Cachexia Sarcopenia and Muscle. 2020;11(1):3-25.
9. Ersoy Y, MacWalter RS, Durmus B, Altay ZE, Baysal O. Predictive Effects of Different Clinical Balance Measures and the Fear of Falling on Falls in Postmenopausal Women Aged 50 Years and Over. Gerontology. 2009;55:660-5.
10. Kang L, Han PP, Wang JZ, Ma YX, Jia LY, Fu LY, et al. Timed Up and Go Test can predict recurrent falls: a longitudinal study of the community-dwelling elderly in China. Clinical Interventions in Aging. 2017;12:2009-16.
11. Savva GM, Donoghue OA, Horgan F, O'Regan C, Cronin H, Kenny RA. Using Timed Up-and-Go to Identify Frail Members of the Older Population. Journals of Gerontology Series a-Biological Sciences and Medical Sciences. 2013;68:441-6.
12. Davis JC, Best JR, Bryan S, Li LC, Hsu CL, Gomez C, et al. Mobility Is a Key Predictor of Change in Well-Being Among Older Adults Who Experience Falls: Evidence From the Vancouver Falls Prevention Clinic Cohort. Archives of physical medicine and rehabilitation. 2015;96(9):1634-40.

13. Doi T, Hirata S, Ono R, Tsutsumimoto K, Misu S, Ando H. The harmonic ratio of trunk acceleration predicts falling among older people: results of a 1-year prospective study. Journal of Neuroengineering and Rehabilitation. 2013;10.
