# Supplementary material for: Predictive Validity of Motor Fitness and Flexibility Tests in Adults and Older Adults: A Systematic Review
Source: J Clin Med. 2022 Jan 10;11(2):328. doi: 10.3390/jcm11020328 (PMC8779466; doi:10.3390/jcm11020328)
Supplement: Supplementary file 1 [file jcm-11-00328-s001.zip › jcm-1466181-supplementary/SupplementaryMaterial1_Search strategy terms.pdf]

Supplementary material S1: Search strategy terms.

PubMed

((((((((((((((Adult[Mesh:NoExp] OR "Middle Aged"[Mesh:NoExp] OR "Aged/classification"[Mesh:NoExp]) AND (“Longitudinal Studies”[Mesh]) AND (“Range of Motion, Articular”[Mesh Major Topic])) OR ((Adult[Mesh:NoExp] OR "Middle Aged"[Mesh:NoExp] OR "Aged/classification"[Mesh:NoExp]) AND (“Longitudinal Studies”[Mesh]) AND ("Postural Balance"[Mesh]))) OR ((Adult[Mesh:NoExp] OR "Middle Aged"[Mesh:NoExp] OR "Aged/classification"[Mesh:NoExp]) AND (“Longitudinal Studies”[Mesh]) AND ("Arteriosclerosis"[Mesh])))) OR ((Adult[Mesh:NoExp] OR "Middle Aged"[Mesh:NoExp] OR "Aged/classification"[Mesh:NoExp]) AND (“Longitudinal Studies”[Mesh]) AND ("Walking Speed"[Mesh]))))) OR ((Adult[Mesh:NoExp] OR "Middle Aged"[Mesh:NoExp] OR "Aged/classification"[Mesh:NoExp]) AND (“Longitudinal Studies”[Mesh]) AND (Agility*[All Fields])))))) OR ((Adult[Mesh:NoExp] OR "Middle Aged"[Mesh:NoExp] OR "Aged/classification"[Mesh:NoExp]) AND (“Longitudinal Studies”[Mesh]) AND ("Accidental Falls"[Mesh]))))))) OR ((Adult[Mesh:NoExp] OR "Middle Aged"[Mesh:NoExp] OR "Aged/classification"[Mesh:NoExp]) AND (“Longitudinal Studies”[Mesh]) AND ("Disabled Persons/prevention and control"[Mesh] OR "Disabled Persons/rehabilitation"[Mesh] OR "Disabled Persons/therapy"[Mesh]) OR (“disability*”) OR ("Mobility Limitation"[Mesh])))))))) OR ((Adult[Mesh:NoExp] OR "Middle Aged"[Mesh:NoExp] OR "Aged/classification"[Mesh:NoExp]) AND (“Longitudinal Studies”[Mesh]) AND ("Frailty"[Mesh]))))))))) OR ((Adult[Mesh:NoExp]

OR "Middle Aged"[Mesh:NoExp] OR "Aged/classification"[Mesh:NoExp]) AND (“Longitudinal Studies”[Mesh]) AND ("Cardiovascular Diseases/adverse effects"[Mesh] OR "Cardiovascular Diseases/analysis"[Mesh] OR "Cardiovascular Diseases/complications"[Mesh] OR "Cardiovascular Diseases/diagnosis"[Mesh] OR "Cardiovascular Diseases/epidemiology"[Mesh] OR "Cardiovascular Diseases/genetics"[Mesh] OR "Cardiovascular Diseases/growth and development"[Mesh] OR "Cardiovascular Diseases/immunology"[Mesh] OR "Cardiovascular Diseases/injuries"[Mesh] OR "Cardiovascular Diseases/metabolism"[Mesh] OR "Cardiovascular Diseases/mortality"[Mesh] OR "Cardiovascular Diseases/physiology"[Mesh] OR "Cardiovascular Diseases/physiopathology"[Mesh] OR "Cardiovascular Diseases/prevention and control"[Mesh] OR "Cardiovascular Diseases/rehabilitation"[Mesh] OR "Cardiovascular Diseases/statistics and numerical data"[Mesh] OR "Cardiovascular Diseases/therapy"[Mesh] OR "Cardiovascular Diseases/transplantation"[Mesh])))))))))) OR ((Adult[Mesh:NoExp] OR "Middle Aged"[Mesh:NoExp] OR "Aged/classification"[Mesh:NoExp]) AND (“Longitudinal Studies”[Mesh]) AND ("Neoplasms"[Mesh] OR “cancer”[All Fields]))))))))))) OR ((Adult[Mesh:NoExp] OR "Middle Aged"[Mesh:NoExp] OR "Aged/classification"[Mesh:NoExp]) AND (“Longitudinal Studies”[Mesh]) AND ("Mortality"[Mesh])))))))))))) OR ((Adult[Mesh:NoExp] OR "Middle Aged"[Mesh:NoExp] OR "Aged/classification"[Mesh:NoExp]) AND (“Longitudinal Studies”[Mesh]) AND ("Dementia"[Mesh] OR "Stress Disorders, Traumatic"[Mesh] OR “stress*” [All Fields])))))))))))))) OR ((Adult[Mesh:NoExp] OR "Middle Aged"[Mesh:NoExp] OR

"Aged/classification"[Mesh:NoExp]) AND (“Longitudinal Studies”[Mesh]) AND ("Quality of Life"[Mesh] OR "well-being*"[All Fields]))))))))))))))

Web of Sciences

((Adult* OR "Middle aged" OR Aged* OR Elderly* AND "Longitudinal Studies" OR "Longitudinal Study" OR "Studies, Longitudinal" OR "Study, Longitudinal" OR "Tuskegee Syphilis Study" OR "Syphilis Studies, Tuskegee" OR "Syphilis Study, Tuskegee" OR "Tuskegee Syphilis Studies" OR "Jackson Heart Study" OR "Heart Studies, Jackson" OR "Heart Study, Jackson" OR "Jackson Heart Studies" OR "Studies, Jackson Heart" OR "California Teachers Study" OR "California Teachers Studies" OR "Studies, California Teachers" OR "Study, California Teachers" OR "Teachers Studies, California" OR "Teachers Study, California" OR "Bogalusa Heart Study" OR "Bogalusa Heart Studies" OR "Heart Studies, Bogalusa" OR "Heart Study, Bogalusa" OR "Studies, Bogalusa Heart" OR "Study, Bogalusa Heart" OR "Framingham Heart Study" OR "Framingham Heart Studies" OR "Heart Studies, Framingham" OR "Heart Study, Framingham" OR "Longitudinal Survey" OR "Longitudinal Surveys" OR "Survey, Longitudinal" OR "Surveys, Longitudinal" OR "Prospective study" OR "Retrospective study" AND "Range of Motion, Articular" OR "Joint Range of Motion" OR "Joint Flexibility" OR "Flexibility, Joint" OR "Range of Motion" OR "Passive Range of Motion" AND "Postural Balance" OR "Balance Postural" OR "Musculoskeletal Equilibrium" OR "Equilibrium, Musculoskeletal" OR "Postural Equilibrium" OR "Equilibrium Postural" AND "Running Speed" OR "Walking Speed" OR "Gait Speed" OR "Walking Pace" AND Agility* AND "Accidental Falls" OR "Falls" OR "Falling" OR "Falls, Accidental" OR

"Accidental Fall" OR "Fall, Accidental" OR "Slip and Fall" OR "Fall and Slip" AND "Frailty" OR "Frailties" OR "Frailness" OR "Frailty Syndrome" OR "Debility" OR "Debilities" AND "Bone Health*" AND "Arteriosclerosis" OR "Arterioscleroses" AND "Low Back Pain" OR "Back Pain, Low" OR "Back Pains, Low" OR "Low Back Pains" OR "Pain, Low Back" OR "Pains, Low Back" OR "Lumbago" OR "Lower Back Pain" OR "Back Pain, Lower" OR "Back Pains, Lower" OR "Lower Back Pains" OR "Pain, Lower Back" OR "Pains, Lower Back" OR "Low Back Ache" OR "Ache, Low Back" OR "Aches, Low Back" OR "Back Ache, Low" OR "Back Aches, Low" OR "Low Back Aches" OR "Low Backache" OR "Backache, Low" OR "Backaches, Low" OR "Low Backaches" OR "Low Back Pain, Postural" OR "Postural Low Back Pain" OR "Low Back Pain, Posterior Compartment" OR "Low Back Pain, Recurrent" OR "Recurrent Low Back Pain" OR "Low Back Pain, Mechanical" OR "Mechanical Low Back Pain" AND Disability* AND "Mobility Limitation" OR "Limitation, Mobility" OR "Mobility Limitations" OR "Ambulation Difficulty" OR "Ambulation Difficulties" OR "Difficulties, Ambulation" OR "Difficulty, Ambulation" OR "Difficulty Ambulation" OR "Ambulatory Difficulty" OR "Ambulatory Difficulties" OR "Difficulties, Ambulatory" OR "Difficulty Walking" OR "Walking, Difficulty" AND "Cardiovascular Risk" OR "Cardiovascular Diseases" AND Cancer* AND "Mortality" OR "Mortalities" OR "Case Fatality Rate" OR "Case Fatality Rates" OR "Rate, Case Fatality" OR "Rates, Case Fatality" OR "Mortality, Excess" OR "Excess Mortalities" OR "Mortalities, Excess" OR "Excess Mortality" OR "Decline, Mortality" OR "Declines, Mortality" OR "Mortality Declines" OR "Mortality Decline" OR "Mortality Determinants" OR "Determinant, Mortality" OR "Mortality Determinant" OR "Determinants, Mortality" OR "Mortality, Differential" OR "Differential Mortalities" OR "Mortalities, Differential" OR "Differential Mortality" OR "Age-Specific Death Rate" OR

"Age-Specific Death Rates" OR "Death Rate, Age-Specific" OR "Death Rates, Age-Specific" OR "Rate, Age-Specific Death" OR "Rates, Age-Specific Death" OR "Age Specific Death Rate" OR "Death Rate" OR "Death Rates" OR "Rate, Death" OR "Rates, Death" OR "Mortality Rate" OR "Mortality Rates" OR "Rate, Mortality" OR "Rates, Mortality" AND "Dementia" OR "Dementias" OR "Amentia" OR "Amentias" OR "Senile Paranoid Dementia" OR "Dementias, Senile Paranoid" OR "Paranoid Dementia, Senile" OR "Paranoid Dementias, Senile" OR "Senile Paranoid Dementias" OR "Familial Dementia" OR "Dementia, Familial" OR "Dementias, Familial" OR "Familial Dementias" AND "Depression" OR "Depressions" OR "Depressive Symptoms" OR "Depressive Symptom" OR "Symptom, Depressive" OR "Symptoms, Depressive" OR "Emotional Depression" OR "Depression, Emotional" OR "Depressions, Emotional" OR "Emotional Depressions" AND "Anxiety" OR "Hypervigilance" OR "Nervousness" OR "Social Anxiety" OR "Anxieties, Social" OR "Anxiety, Social" OR "Social Anxieties" AND Stress* AND "Quality of Life" OR "Life Quality" OR "Health-Related Quality Of Life" OR "Health Related Quality Of Life" OR "HRQOL" OR "QOL" OR "Well-Being*"))
