# Supplementary material for: Predictive Validity of Motor Fitness and Flexibility Tests in Adults and Older Adults: A Systematic Review
Source: J Clin Med. 2022 Jan 10;11(2):328. doi: 10.3390/jcm11020328 (PMC8779466; doi:10.3390/jcm11020328)
Supplement: Supplementary file 1 [file jcm-11-00328-s001.zip › jcm-1466181-supplementary/SupplementaryMaterial2_ListofExcluded.pdf]

Supplementary material S2: List of excluded studies

1. Amigues I, Schott AM, Amine M, Gelas-Dore B, Veerabudun K, Paillaud E, et al. Low Skeletal Muscle Mass and Risk of Functional Decline in Elderly Community-Dwelling Women: The Prospective EPIDOS Study. Journal of the American Medical Directors Association. 2013;14:352-7.

2. Analan PD, Yilmaz EE, Leblebici B. Evaluation of postural balance and risk of fall in a healthy adult population. Cukurova Medical Journal. 2016;41(2):236-41.

3. Aoyagi Y, Park H, Watanabe E, Park S, Shephard RJ. Habitual Physical Activity and Physical Fitness in Older Japanese Adults: The Nakanojo Study. Gerontology. 2009;55:523-31.

4. Artaud F, Singh-Manoux A, Dugravot A, Tzourio C, Elbaz A. Decline in Fast Gait Speed as a Predictor of Disability in Older Adults. Journal of the American Geriatrics Society. 2015;63:1129-36.

5. Avila-Funes JA, Gray-Donald K, Payette H. Association of Nutritional Risk and Depressive Symptoms with Physical Performance in the Elderly: The Quebec Longitudinal Study of Nutrition as a Determinant of Successful Aging (NuAge). Journal of the American College of Nutrition. 2008;27(4):492-8.

6. Batsis JA, Germain CM, Vasquez E, Zbehlik AJ, Bartels SJ. Physical Activity Predicts Higher Physical Function in Older Adults: The Osteoarthritis Initiative. Journal of Physical Activity & Health. 2016;13:6-16.

7. Beauchet O, Sekhon H, Schott AM, Rolland Y, Muir-Hunter S, Markle-Reid M, et al. Motoric Cognitive Risk Syndrome and Risk for Falls, Their Recurrence, and Postfall Fractures: Results From a Prospective Observational Population-Based Cohort Study. Journal of the American Medical Directors Association. 2019;20(10):1268-73.

8. Bouillon K, Batty GD, Hamer M, Sabia S, Shipley MJ, Britton A, et al. Cardiovascular disease risk scores in identifying future frailty: the Whitehall II prospective cohort study. Heart. 2013;99:737-42.

9. Bowen ME, Crenshaw J, Stanhope SJ. Balance ability and cognitive impairment influence sustained walking in an assisted living facility. Archives of Gerontology and Geriatrics. 2018;77:133-41.

10. Brenowitz WD, Hubbard RA, Crane PK, Gray SL, Zaslavsky O, Larson EB. Longitudinal Associations between Self-Rated Health and Performance-Based Physical Function in a Population-Based Cohort of Older Adults. Plos One. 2014;9.

11. Buchman AS, Boyle PA, Wilson RS, Leurgans S, Shah RC, Bennett DA. Respiratory Muscle Strength Predicts Decline in Mobility in Older Persons. Neuroepidemiology. 2008;31:174-80.

12. Bullain SS, Corrada MM, Shah BA, Mozaffar FH, Panzenboeck M, Kawas CH. Poor Physical Performance and Dementia in the Oldest Old The 90+ Study. Jama Neurology. 2013;70:107-13.

13. Buracchio TJ, Mattek NC, Dodge HH, Hayes TL, Pavel M, Howieson DB, et al. Executive function predicts risk of falls in older adults without balance impairment. BMC geriatrics. 2011;11:74.

14. Cabrero-Garcia J, Munoz-Mendoza CL, Cabanero-Martinez MJ, Gonzalez-Llopis L, Ramos-Pichardo JD, Reig-Ferrer A. Short Physical Performance Battery reference values for patients 70 years-old and over in primary health care. Atencion Primaria. 2012;44(9):540-8.

15. Cano C, Samper-Ternent R, Al Snih S, Markides K, Ottenbacher KJ. Frailty and cognitive impairment as predictors of mortality in older Mexican Americans. Journal of Nutrition Health & Aging. 2012;16:142-7.

16. Cardon-Verbecq C, Loustau M, Guitard E, Bonduelle M, Delahaye E, Koskas P, et al. Predicting falls with the cognitive timed up-and-go dual task in frail older patients. Annals of Physical and Rehabilitation Medicine. 2017;60:83-6.

17. Carrer P, Trevisan C, Curreri C, Giantin V, Maggi S, Crepaldi G, et al. Semmes-Weinstein Monofilament Examination for Predicting Physical Performance and the Risk of Falls in Older People: Results of the Pro.V.A. Longitudinal Study. Arch Phys Med Rehabil. 2018;99(1):137-43 e1.

18. Cesari M, Onder G, Zamboni V, Manini T, Shorr RI, Russo A, et al. Physical function and self-rated health status as predictors of mortality: results from longitudinal analysis in the ilSIRENTE study. BMC geriatrics. 2008;8:34.

19. Chen S, Honda T, Narazaki K, Chen T, Kishimoto H, Haeuchi Y, et al. Physical Frailty is Associated with Longitudinal Decline in Global Cognitive Function in Non-Demented Older Adults: A Prospective Study. Journal of Nutrition Health & Aging. 2018;22:82-8.

20. Chiaranda G, Bernardi E, Codeca L, Conconi F, Myers J, Terranova F, et al. Treadmill walking speed and survival prediction in men with cardiovascular disease: a 10-year follow-up study. Bmj Open. 2013;3.

21. Choi HC, Son KY, Cho B, Park SM, Cho SI. An implication of the short physical performance battery (SPPB) as a predictor of abnormal pulmonary function in aging people. Archives of Gerontology and Geriatrics. 2012;54:448-52.

22. Chu LW, Chi I, Chiu AYY. Incidence and predictors of falls in the Chinese elderly. Annals Academy of Medicine Singapore. 2005;34(1):60-72.

23. Crow RS, Lohman MC, Pidgeon D, Bruce ML, Bartels SJ, Batsis JA. Frailty Versus Stopping Elderly Accidents, Deaths and Injuries Initiative Fall Risk Score: Ability to Predict Future Falls. Journal of the American Geriatrics Society. 2018;66:577-83.

24. DargentMolina P, Favier F, Grandjean H, Baudoin C, Schott AM, Hausherr E, et al. Fall-related factors and risk of hip fracture: The EPIDOS prospective study. Lancet. 1996;348(9021):145-9.

25. Desai A, Goodman V, Kapadia N, Shay BL, Szturm T. Relationship Between Dynamic Balance Measures and Functional Performance in Community-Dwelling Elderly People. Physical Therapy. 2010;90:748-60.

26. Deshpande N, Metter EJ, Bandinelli S, Guralnik J, Ferrucci L. Gait speed under varied challenges and cognitive decline in older persons: a prospective study. Age and Ageing. 2009;38:509-14.

27. Doi T, Shimada H, Makizako H, Tsutsumimoto K, Hotta R, Nakakubo S, et al. Mild Cognitive Impairment, Slow Gait, and Risk of Disability: A Prospective Study. Journal of the American Medical Directors Association. 2015;16:1082-6.

28. Doi T, Tsutsumimoto K, Nakakubo S, Kim MJ, Kurita S, Shimada H. Rethinking the Relationship Between Spatiotemporal Gait Variables and Dementia: A Prospective Study. Journal of the American Medical Directors Association. 2019;20(7):899-903.

29. Donoghue OA, Jansen S, Dooley C, De Rooij S, Van Der Velde N, Kenny RA. Atrial Fibrillation Is Associated With Impaired Mobility in Community-Dwelling Older Adults. Journal of the American Medical Directors Association. 2014;15:929-33.

30. Donoghue OA, Savva GM, Cronin H, Kenny RA, Horgan NF. Using Timed Up and Go and Usual Gait Speed to Predict Incident Disability in Daily Activities Among Community-Dwelling Adults Aged 65 and Older. Archives of Physical Medicine and Rehabilitation. 2014;95:1954-61.

31. Duan-Porter W, Vo TN, Ullman K, Langsetmo L, Strotmeyer ES, Taylor BC, et al. Hospitalization-Associated Change in Gait Speed and Risk of Functional Limitations for Older Adults. Journals of Gerontology Series a-Biological Sciences and Medical Sciences. 2019;74(10):1657-63.

32. Dumurgier J, Artaud F, Touraine C, Rouaud O, Tavernier B, Dufouil C, et al. Gait Speed and Decline in Gait Speed as Predictors of Incident Dementia. Journals of Gerontology Series a-Biological Sciences and Medical Sciences. 2017;72:655-61.

33. Egerton T, Paterson K, Helbostad JL. The Association Between Gait Characteristics and Ambulatory Physical Activity in Older People: A Cross-Sectional and Longitudinal Observational Study Using Generation 100 Data. Journal of Aging and Physical Activity. 2017;25:10-9.

34. Ekedahl H, Jönsson B, Frobell RB. Fingertip-to-floor test and straight leg raising test: validity, responsiveness, and predictive value in patients with acute/subacute low back pain. Archives of physical medicine and rehabilitation. 2012;93:2210-5.

35. Elbaz A, Shipley MJ, Nabi H, Brunner EJ, Kivimaki M, Singh-Manoux A. Trajectories of the Framingham general cardiovascular risk profile in midlife and poor

motor function later in life: The Whitehall II study. International Journal of Cardiology. 2014;172:96-102.

36. Ensrud KE, Lui LY, Paudel ML, Schousboe JT, Kats AM, Cauley JA, et al. Effects of Mobility and Cognition on Risk of Mortality in Women in Late Life: A Prospective Study. Journals of Gerontology Series a-Biological Sciences and Medical Sciences. 2016;71:759-65.

37. Era P, Rantanen T. Changes in physical capacity and sensory/psychomotor functions from 75 to 80 years of age and from 80 to 85 years of age - A longitudinal study. Scandinavian Journal of Social Medicine. 1997:25-43.

38. Gillain S, Boutaayamou M, Schwartz C, Dardenne N, Bruyere O, Bruls O, et al. Gait symmetry in the dual task condition as a predictor of future falls among independent older adults: a 2-year longitudinal study. Aging Clinical and Experimental Research. 2019;31(8):1057-67.

39. Gulich M, Zeitler HP. Walking and counting test for assessing the risk of falling in the elderly. Deutsche Medizinische Wochenschrift. 2000;125(9):245-8.

40. Gutierrez-Misis A, Sanchez-Santos MT, Banegas JR, Castell MV, Gonzalez-Montalvo JI, Otero A. Walking speed and high blood pressure mortality risk in a spanish elderly population. Journal of Human Hypertension. 2015;29:566-72.

41. Hackett RA, Davies-Kershaw H, Cadar D, Orrell M, Steptoe A. Walking Speed, Cognitive Function, and Dementia Risk in the English Longitudinal Study of Ageing. Journal of the American Geriatrics Society. 2018;66:1670-5.

42. Hatayama T, Nagano M, Une H, Yoshitake Y, Kimura Y, Momose Y, et al. THE ASSOCIATION BETWEEN INCIDENCE OF FALLS AND PHYSICAL FITNESS IN COMMUNITY- DWELLING ELDERLY. Japanese Journal of Physical Fitness and Sports Medicine. 2008;57(4):503-10.

43. Hausdorff JM, Rios DA, Edelberg HK. Gait variability and fall risk in community-living older adults: A 1-year prospective study. Archives of Physical Medicine and Rehabilitation. 2001;82:1050-6.

44. Hebert LE, Bienias JL, McCann JJ, Scherr PA, Wilson RS, Evans DA. Upper and lower extremity motor performance and functional impairment in Alzheimer's disease. American journal of Alzheimer's disease and other dementias. 2010;25(5):425-31.

45. Heiland EG, Qiu CX, Wang R, Santoni G, Liang Y, Fratiglioni L, et al. Cardiovascular Risk Burden and Future Risk of Walking Speed Limitation in Older Adults. Journal of the American Geriatrics Society. 2017;65:2418-24.

46. Heiland EG, Welmer A-K, Wang R, Santoni G, Angleman S, Fratiglioni L, et al. Association of mobility limitations with incident disability among older adults: a population-based study. Age and ageing. 2016;45:812-9.

47. Hirani V, Blyth F, Naganathan V, Le Couteur DG, Seibel MJ, Waite LM, et al. Sarcopenia Is Associated With Incident Disability, Institutionalization, and Mortality in Community-Dwelling Older Men: The Concord Health and Ageing in Men Project. Journal of the American Medical Directors Association. 2015;16(7):607-13.

48. Hsu CL, Liang CK, Liao MC, Chou MY, Lin YT. Slow gait speed as a predictor of 1-year cognitive decline in a veterans' retirement community in southern Taiwan. Geriatrics & Gerontology International. 2017;17:14-9.

49. Inzitari M, Metti A, Rosano C, Udina C, Perez LM, Carrizo G, et al. Qualitative neurological gait abnormalities, cardiovascular risk factors and functional status in older community-dwellers without neurological diseases: The Healthy Brain Project. Experimental Gerontology. 2019;124.

50. Ishizaki T, Kobayashi E, Fukaya T, Takahashi Y, Shinkai S, Liang J. Association of physical performance and self-rated health with multimorbidity among older adults: Results from a nationwide survey in Japan. Archives of Gerontology and Geriatrics. 2019;84.

51. Jonkman NH, Del Panta V, Hoekstra T, Colpo M, van Schoor NM, Bandinelli S, et al. Predicting Trajectories of Functional Decline in 60- to 70-Year-Old People. Gerontology. 2018;64:212-21.

52. Kikkert LHJ, Vuillerme N, van Campen JP, Hortobagyi T, Lamoth CJ. Walking ability to predict future cognitive decline in old adults: A scoping review. Ageing Research Reviews. 2016;27:1-14.

53. Kirkness CS, Ren J. Race Differences: Use of Walking Speed to Identify Community-Dwelling Women at Risk for Poor Health Outcomes-Osteoarthritis Initiative Study. Physical Therapy. 2015;95:955-65.

54. Konno K, Katsumata Y, Arai A, Tamashiro H. Functional status and active life expectancy among senior citizens in a small town in Japan. Archives of Gerontology and Geriatrics. 2004;38(2):153-66.

55. Kuate-Tegueu C, Avila-Funes JA, Simo N, Le Goff M, Amieva H, Dartigues JF, et al. Association of Gait Speed, Psychomotor Speed, and Dementia. Journal of Alzheimers Disease. 2017;60:585-92.

56. Kulmala J, Ngandu T, Pajala S, Lehtisalo J, Levalahti E, Antikainen R, et al. Leisure-Time and Occupational Physical Activity in Early and Late Adulthood in Relation to Later Life Physical Functioning. Journal of Physical Activity & Health. 2016;13:1079-87.

57. Lane NE, Stukel TA, Boyd CM, Wodchis WP. Long-Term Care Residents' Geriatric Syndromes at Admission and Disablement Over Time: An Observational Cohort Study. The journals of gerontology Series A, Biological sciences and medical sciences. 2019;74(6):917-23.

58. Lang IA, Llewellyn DJ, Alexander K, Melzer D. Obesity, physical function, and mortality in older adults. J Am Geriatr Soc. 2008;56(8):1474-8.

59. Leach JM, Mancini M, Kaye JA, Hayes TL, Horak FB. Day-to-Day Variability of Postural Sway and Its Association With Cognitive Function in Older Adults: A Pilot Study. Frontiers in Aging Neuroscience. 2018;10.

60. Lipsitz LA, Manor B, Habtemariam D, Iloputaife I, Zhou JH, Travison TG. The pace and prognosis of peripheral sensory loss in advanced age: association with gait speed and falls. BMC geriatrics. 2018;18.

61. Looijaard S, Slee-Valentijn MS, Groeneveldt LN, Deeg DJH, Huisman M, Maier AB. Do older individuals who are diagnosed with cancer have worse physical performance prior to diagnosis compared to matched controls? A longitudinal cohort study. BMC geriatrics. 2018;18.

62. MacDonald SWS, Hundza S, Love JA, DeCarlo CA, Halliday DWR, Brewster PWH, et al. Concurrent Indicators of Gait Velocity and Variability Are Associated with 25-Year Cognitive Change: A Retrospective Longitudinal Investigation. Frontiers in Aging Neuroscience. 2017;9.

63. Marquis S, Moore MM, Howieson DB, Sexton G, Payami H, Kaye JA, et al. Independent predictors of cognitive decline in healthy elderly persons. Archives of Neurology. 2002;59(4):601-6.

64. Martins AC, Moreira J, Silva C, Silva J, Tonelo C, Baltazar D, et al. Multifactorial Screening Tool for Determining Fall Risk in Community-Dwelling Adults Aged 50 Years or Over (FallSensing): Protocol for a Prospective Study. Jmir Research Protocols. 2018;7.

65. Melzer D, Lan TY, Guralnik JM. The predictive validity for mortality of the index of mobility-related limitation - results from the EPESE study. Age and Ageing. 2003;32(6):619-25.

66. Metti AL, Best JR, Shaaban CE, Ganguli M, Rosano C. Longitudinal changes in physical function and physical activity in older adults. Age and Ageing. 2018;47:558-64.

67. Mielke MM, Roberts RO, Savica R, Cha R, Drubach DI, Christianson T, et al. Assessing the Temporal Relationship Between Cognition and Gait: Slow Gait Predicts Cognitive Decline in the Mayo Clinic Study of Aging. Journals of Gerontology Series a-Biological Sciences and Medical Sciences. 2013;68:929-37.

68. Montero-Odasso M, Sarquis-Adamson Y, Song HY, Bray NW, Pieruccini-Faria F, Speechley M. Polypharmacy, Gait Performance, and Falls in Community-Dwelling Older Adults. Results from the Gait and Brain Study. Journal of the American Geriatrics Society. 2019;67(6):1182-8.

69. Morley JE, Malmstrom TK, Miller DK. A simple frailty questionnaire (FRAIL) predicts outcomes in middle aged African Americans. Journal of Nutrition Health & Aging. 2012;16:601-8.

70. Nieves JW, Li T, Zion M, Gussekloo J, Pahor M, Bernabei R, et al. The clinically meaningful change in physical performance scores in an elderly cohort. Aging Clinical and Experimental Research. 2007;19(6):484-91.

71. Oppewal A, Hilgenkamp TIM, van Wijck R, Schoufour JD, Evenhuis HM. Physical fitness is predictive for a decline in the ability to perform instrumental activities of daily living in older adults with intellectual disabilities: Results of the HA-ID study. Research in Developmental Disabilities. 2015;41-42:76-85.

72. Ostir GV, Berges IM, Ottenbacher KJ, Fisher SR, Barr E, Hebel JR, et al. Gait Speed and Dismobility in Older Adults. Archives of Physical Medicine and Rehabilitation. 2015;96:1641-5.

73. Owusu C, Margevicius S, Schluchter M, Koroukian SM, Berger NA. Short Physical Performance Battery, usual gait speed, grip strength and Vulnerable Elders Survey each predict functional decline among older women with breast cancer. Journal of Geriatric Oncology. 2017;8:356-62.

74. Patience J, Lai KSP, Russell E, Vasudev A, Montero-Odasso M, Burhan AM. Relationship Between Mood, Thinking, and Walking: A Systematic Review Examining Depressive Symptoms, Executive Function, and Gait. American Journal of Geriatric Psychiatry. 2019;27(12):1375-83.

75. Pinto JM, Wroblewski KE, Huisingh-Scheetz M, Correia C, Lopez KJ, Chen RC, et al. Global Sensory Impairment Predicts Morbidity and Mortality in Older US Adults. Journal of the American Geriatrics Society. 2017;65:2587-95.

76. Preto LSR, Santos ALN, Mendes ME, Novo AP, Pimentel M. Functional impairment, fear of falling and body composition in institutionalized elderly. Enfermeria Clinica. 2015;25(2):81-6.

77. Prudon I, Noyez L, Van Swieten H, Scheffer GJ. Is gait speed improving performance of the EuroSCORE II for prediction of early mortality and major morbidity in the elderly? Journal of Cardiovascular Surgery. 2016;57(4):592-7.

78. Read S, Grundy E. Allostatic Load and Health in the Older Population of England: A Crossed-Lagged Analysis. Psychosomatic Medicine. 2014;76:490-6.

79. Ribeiro SML, Morley JE, Malmstrom TK, Miller DK. Fruit and vegetable intake and physical activity as predictors of disability risk factors in African-American middle-aged individuals. Journal of Nutrition Health & Aging. 2016;20:891-6.

80. Sanders JB, Bremmer MA, Comijs HC, Deeg DJH, Beekman ATF. Gait Speed and the Natural Course of Depressive Symptoms in Late Life; An Independent Association With Chronicity? Journal of the American Medical Directors Association. 2016;17:331-5.

81. Seo M, Won CW, Kim S, Yoo JH, Kim YH, Kim BS. The Association of Gait Speed and Frontal Lobe among Various Cognitive Domains: The Korean Frailty and Aging Cohort Study (KFACS). Journal of Nutrition Health & Aging. 2020;24(1):91-7.

82. Shea CA, Ward RE, Welch SA, Kiely DK, Goldstein R, Bean JF. Inability to Perform the Repeated Chair Stand Task Predicts Fall-Related Injury in Older Primary Care Patients. American Journal of Physical Medicine & Rehabilitation. 2018;97:426-32.

83. Simonsick EM, Guralnik JM, Volpato S, Balfour J, Fried LP. Just get out the door! Importance of walking outside the home for maintaining mobility: Findings from the Women's Health and Aging Study. Journal of the American Geriatrics Society. 2005;53:198-203.

84. Sonn U, Frandin K, Grimby G. INSTRUMENTAL ACTIVITIES OF DAILY LIVING-RELATED TO IMPAIRMENTS AND FUNCTIONAL LIMITATIONS IN 70-YEAR-OLDS AND CHANGES BETWEEN 70 AND 76 YEARS OF AGE. Scandinavian Journal of Rehabilitation Medicine. 1995;27(2):119-28.

85. Sourdet S, Van Kan GA, Soto ME, Houles M, Cantet C, Nourhashemi F, et al. Prognosis of an Abnormal One-Leg Balance in Community-Dwelling Patients With Alzheimer's Disease: A 2-Year Prospective Study in 686 Patients of the REAL.FR Study. Journal of the American Medical Directors Association. 2012;13.

86. Stevens KN, Lang IA, Guralnik JM, Melzer D. Epidemiology of balance and dizziness in a national population: findings from the English Longitudinal Study of Ageing. Age Ageing. 2008;37(3):300-5.

87. Sugiura M, Nagasaki H, Furuna T, Okuzumi H. Walking ability of older adults in the community - A four-year follow-up study. Japanese Journal of Physical Fitness and Sports Medicine. 1998;47:443-52.

88. Suttanon P, Hill KD, Said CM, Dodd KJ. A longitudinal study of change in falls risk and balance and mobility in healthy older people and people with Alzheimer disease. Am J Phys Med Rehabil. 2013;92(8):676-85.

89. Taniguchi Y, Yoshida H, Fujiwara Y, Motohashi Y, Shinkai S. A Prospective Study of Gait Performance and Subsequent Cognitive Decline in a General Population of Older Japanese. Journals of Gerontology Series a-Biological Sciences and Medical Sciences. 2012;67:796-803.

90. Tian Q, An Y, Resnick SM, Studenski S. The relative temporal sequence of decline in mobility and cognition among initially unimpaired older adults: Results from the Baltimore Longitudinal Study of Aging. Age and Ageing. 2017;46:445-51.

91. Tomey K, Sowers MR, Harlow S, Jannausch M, Zheng HY, Bromberger J. Physical functioning among mid-life women: Associations with trajectory of depressive symptoms. Social Science & Medicine. 2010;71:1259-67.

92. Vasunilashorn S, Coppin AK, Patel KV, Lauretani F, Ferrucci L, Bandinelli S, et al. Use of the Short Physical Performance Battery Score to predict loss of ability to walk 400 meters: analysis from the InCHIANTI study. The journals of gerontology Series A, Biological sciences and medical sciences. 2009;64(2):223-9.

93. Vermeulen J, Neyens JCL, van Rossum E, Spreeuwenberg MD, de Witte LP. Predicting ADL disability in community-dwelling elderly people using physical frailty indicators: a systematic review. BMC geriatrics. 2011;11.

94. Welmer AK, Rizzuto D, Qiu CX, Caracciolo B, Laukka EJ. Walking Speed, Processing Speed, and Dementia: A Population-Based Longitudinal Study. Journals of Gerontology Series a-Biological Sciences and Medical Sciences. 2014;69:1503-10.

95. Woo J, Ho SC, Yu ALM. Walking speed and stride length predicts 36 months dependency, mortality, and institutionalization in Chinese aged 70 and older. Journal of the American Geriatrics Society. 1999;47(10):1257-60.

96. Wu LW, Chen WL, Peng TC, Chiang ST, Yang HF, Sun YS, et al. All-cause mortality risk in elderly individuals with disabilities: a retrospective observational study. Bmj Open. 2016;6.

97. Zhang L, Guo LW, Wu HT, Gong XW, Lv JQ, Yang YF. Role of physical performance measures for identifying functional disability among Chinese older adults: Data from the China Health and Retirement Longitudinal Study. Plos One. 2019;14(4).
