# Supplementary material for: Predictive Validity of Motor Fitness and Flexibility Tests in Adults and Older Adults: A Systematic Review
Source: J Clin Med. 2022 Jan 10;11(2):328. doi: 10.3390/jcm11020328 (PMC8779466; doi:10.3390/jcm11020328)
Supplement: Supplementary file 1 [file jcm-11-00328-s001.zip › jcm-1466181-supplementary/SupplementaryTable 6.pdf]

Supplementary Table S6. Systematic reviews/meta-analysis on predictive validity of motor fitness tests for adverse health outcomes in adults and older adults.

Author	Type of review; number of studies (k)	Sample age	Fitness tests	Health outcomes	Main results	Conclusions
**Falls**						
Cavanaugh et al. 2018 [1]	Systematic review K = 3	≥60	Gait speed (6m) Dynamic balance (functional reach test, cm (FRT)) Timed Up&Go (TUG)	Falls	Slower gait speed (≥6s in total): RR=1.8 (1.2-2.6). FRT: r=0.013, *p*>0.05. TUG: r=0.143, *p*>0.05.	Slower gait speed identified falls. Neither the FRT nor TUG test and falls risk were significant.
Van Kan et al. 2009 [2]	Systematic review K = 4	≥65	Gait speed (5m, 6m and 10m)	Falls	Slower gait speed (<0.67m/s): RR ranging from 0.2 to 5.4 (0.1-14.3).	Slow gait speed was an independent predictor of falls.
**Cognitive decline and impairment**						
Quan et al. 2017 [3]	Meta-analysis K = 17	≥60	Gait speed (2.4m, 3m, 4m, 4.9m, 6m, 7m, 8m, 9m, 10m, 60m and 400m)	Cognitive decline and dementia	Slower gait speed: RR=1.9 (1.5–2.3), for cognitive decline. Slower gait speed: RR=1.7 (1.4–1.9), for dementia.	Slow or decreased gait speed predicted elevated risk of cognitive decline and dementia.

					Every 1 m/s decrement in gait speed: RR=1.1 (1.1–1.2), for dementia.	
Van Kan et al. 2009 [2]	Systematic review K = 7	≥65	Gait speed (2.4m, 4m, 4.6m, 5m and 6m)	Cognitive decline	Slower gait speed (<1m/s): RR ranging from 0.4 to 5.6 (0.2-12.6).	Gait speed at usual pace predicted cognitive impairment.
Grande et al. 2019 [4]	Systematic review K = 39	62-93	Gait speed (2.4m, 3m, 4m, 4.5m, 4.8m, 5m, 5.6m, 6m, 7m, 7.6m, 9m, 15m, 40m)	Cognitive decline and/or dementia	Slower gait speed (<0.8m/s): RR ranging from 1.1 to 5.0 (0.53-48.2).	Slow or decreased gait speed predicted elevated risk of cognitive decline and/or dementia.
Peel et al. 2019 [5]	Systematic review K = 36 Meta-analysis K = 27	≥60	Gait speed (2.4m, 3.7m, 4m, 4.6m, 5m, 5.3m, 5.6m, 6m, 7m, 7.6m, 7.9m, 8m, 10m, 20m, 30m)	Cognitive decline and dementia	Parameter estimate=-0.1 (-0.1, -0.1) m/s, *p*<0.001, for cognitive decline. Parameter estimate=-0.2 (-0.2, -0.2) m/s, *p*<0.001, for mild dementia. Parameter estimate=-0.4 (-0.5, -0.3) m/s, *p*<0.001, for moderate dementia.	Slow or decreased gait speed predicted elevated risk of cognitive decline and dementia.
**Mobility limitations and disability**						
Cavanaugh et al. 2018 [1]	Systematic review K = 6	≥60		Disability in instrumental daily activities	Usual gait speed (<1m/s): HR=1.5 (1.3-1.7), for persistent mobility disability; and: HR=1.5 (1.2-	Slower gait speed was a predictor

		Gait speed (2.4m, 4m, 6m and 15.24m)	(IADL); mobility limitations	1.8), for persistent severe mobility disability.	of disability in IADL and mobility limitations.
				Slower gait speed, 2.4m (≥9s in total): OR=3.4 (1.8–6.5); 6-8s: OR=2.6 (1.4–4.9); 4-5s: OR=2.1 (1.2–4.0), for mobility limitations.	
				Fastest gait speed: OR=7.9, *p*=0.04, for mobility limitations.	
		Postural balance (semi-tandem stance, tandem stance one-leg stance, 30s/test (0-90s); side-by-side, semi-tandem stance, tandem stance, 10s/test)		Gait speed, 2.4m (≥9s in total): OR=5.4 (1.2–23.6), for IADL.	Balance tests identified preclinical disability due to disability in IADL and mobility disability.
				Gait speed, 4m (<0.6m/s): 12 months, OR=0.5 (0.02-16.00); 18 months, OR=0.2 (0.01-8.79), for IADL.	
		TUG		Total balance test (<53s): HR=1.6 (1.4-1.8), for persistent mobility disability; and HR=1.85 (1.6-2.2), for persistent severe mobility disability.	The tug test did not identify preclinical disability in mobility and IADL.
				Tandem stance (>2-<10s): OR=1.6 (1.1-2.6), for mobility disability.	

					Tandem stance (>2-<10s): OR=2.5 (1.2-4.8), for IADL.<br>TUG: OR=1.1, *p*=0.24, for mobility disability.	
Van Kan et al. 2009 [2]	Systematic review<br>K = 10	≥65	Gait speed (2.4m, 4m, 4.6m, 4.9m, 6m, 11m and 400m)	IADL; mobility limitations	Slower gait speed (<1m/s): RR ranging from 0.7 to 6.1 (0.5-23.6), for IADL.<br>Slower gait speed (<1m/s): RR ranging from 0.6 to 2.1 (0.9-6.5), for mobility limitations.	Slow gait speed was found to predict disability in IADL and mobility limitations.
Wang et al. 2020 [6]	Systematic review<br>K = 62<br>Meta-analysis<br>K = 45	54-86	Gait speed (*unreported distances*)<br>Postural balance (Berg Balance Scale, one-leg stance (5s), side-by-side, semi-tandem stance, tandem stance)<br>TUG	IADL	Slower gait speed (<1m/s): OR=4.4 (1.3–14.5).<br>One-leg balance stance (<5s): OR=2.7 (1.3–5.7).<br>Worse TUG performance (>12s): OR=3.4 (1.9–6.3).	Worse physical performance in gait speed, one-leg balance or TUG predicted IADL.
**Hospitalization/ institutionalisation**						
Cavanaugh et al. 2018 [1]	Systematic review<br>K = 2	≥60	Gait speed (6m)<br>Postural balance ((semi-tandem stance, tandem	Hospitalization	Slower gait speed (<1m/s): HR=1.3 (1.0–1.6).	Slow gait speed and impairment balance identified hospitalization.

			stance one-leg stance, 30s/test (0-90s))		Total balance (<53s): HR=1.4 (1.1-1.7).	
Van Kan et al. 2009 [2]	Systematic review K = 5	≥65	Gait speed (4m, 4.9m, 6m, 10m)	Institutionalisatio n or hospitalization	Slower gait speed (<1m/s): RR ranging from 1.0 to 5.9 (1.0-18.5).	Slow gait speed was found to predict institutionalisation or hospitalization.
**Mortality**						
Van Kan et al. 2009 [2]	Systematic review K = 9	≥65	Gait speed (2.4m, 4m, 4.6m, 4.9m and 6m)	All-cause mortality	Slower gait speed (<1m/s): RR ranging from 0.7 to 7.4 (0.5-14.5).	Slow gait speed was found to predict mortality.

B, non-standardized regression coefficient; FRT, Functional Reach Test; HR, Hazard Ratio; IADL, Disability in Instrumental Activities of Daily Living; OR, Odd Ratio; RR, relative risk; TUG, Timed Up&Go test.

REFERENCES

1. Cavanaugh EJ, Richardson J, McCallum CA, Wilhelm M. The Predictive Validity of Physical Performance Measures in Determining Markers of Preclinical Disability in Community-Dwelling Middle-Aged and Older Adults: A Systematic Review. Physical Therapy. 2018;98(12):1010-21.
2. van Kan GA, Rolland Y, Andrieu S, Bauer J, Beauchet O, Bonnefoy M, et al. GAIT SPEED AT USUAL PACE AS A PREDICTOR OF ADVERSE OUTCOMES IN COMMUNITY-DWELLING OLDER PEOPLE AN INTERNATIONAL ACADEMY ON NUTRITION AND AGING (IANA) TASK FORCE. Journal of Nutrition Health & Aging. 2009;13(10):881-9.
3. Quan MH, Xun PC, Chen C, Wen J, Wang YY, Wang R, et al. Walking Pace and the Risk of Cognitive Decline and Dementia in Elderly Populations: A Meta-analysis of Prospective Cohort Studies. Journals of Gerontology Series a-Biological Sciences and Medical Sciences. 2017;72(2):266-70.
4. Grande G, Triolo F, Nuara A, Welmer AK, Fratiglioni L, Vetrano DL. Measuring gait speed to better identify prodromal dementia. Experimental Gerontology. 2019;124.
5. Peel NM, Alapatt LJ, Jones LV, Hubbard RE. The Association Between Gait Speed and Cognitive Status in Community-Dwelling Older People: A Systematic Review and Meta-analysis. Journals of Gerontology Series a-Biological Sciences and Medical Sciences. 2019;74(6):943-8.
6. Wang DXM, Yao J, Zirek Y, Reijnierse EM, Maier AB. Muscle mass, strength, and physical performance predicting activities of daily living: a meta-analysis. Journal of Cachexia Sarcopenia and Muscle. 2020;11(1):3-25.
