# Supplementary material for: Predictive Validity of Motor Fitness and Flexibility Tests in Adults and Older Adults: A Systematic Review
Source: J Clin Med. 2022 Jan 10;11(2):328. doi: 10.3390/jcm11020328 (PMC8779466; doi:10.3390/jcm11020328)
Supplement: Supplementary file 1 [file jcm-11-00328-s001.zip › jcm-1466181-supplementary/SupplementaryTables 1-5.pdf]

Supplementary Table S1. Longitudinal validity of motor fitness and flexibility tests for falls and hip fracture risk in adults and older adults.

Author	Participants	Sample age, years (Range)	Follow-up	Fitness test	Health outcomes	Main outcomes and conclusions
Kang et al. 2017 [1]	Females= 307 Males= 234	67.4±5.6 (60-86)	1 year	Gait speed (4m)	Falls	Slower gait speed (<0.9m/s): HR= 1.00 (0.99–1.01). The 4m gait test was not associated with falls.
Quach et al. 2011 [2]	Females= 487 Males= 276	78.1±5.4	1.5 years	Gait speed (4m)	Falls	Slower gait speed (<0.6m/s), indoor falls: IRR=2.17 (1.33–3.55). Faster gait speed, outdoor falls: IRR=2.11 (1.40–3.16). There was a nonlinear, U-shaped relationship between gait speed and falls. Those with faster and slower gait speeds were at highest risk of falls.
Callisaya et al. 2016 [3]	Females= 266 Males= 243	74.9±6.8 (60-105)	2 years	Gait speed (4.2m, 7m, 8.5m)	Falls	Slower gait speed (<0.8m/s): RR=1.30 (1.14-1.47). Slower gait speed predicted falls.
Luukinen et al. 1995 [4]	Females= 640 Males= 376	76.1±4.9 (70-92)	2 years	Step length (5m)	Falls	Short step length (<0.45m): RR=1.6 (1.04-2.46). A shorter step length predicted risk of falling.
Abu et al. 2018 [5]	Females= 180 Males=145	67.67±5.5 (60-89)	½ year	Gait speed (6m)	Falls	Slower gait speed: Mean (SD)= 1.06 (0.25), *p*=0.20. The 6m gait speed test was not associated with falls.
Kwan et al. 2012 [6]	Females= 120 Males= 160	74.9±6.4 (65–91)	2 years	Gait speed (6m)	Falls	Slower gait speed: IRR= 0.95 (0.60–0.94). Slower gait speed did not predict falls.
Muraki et al. 2013 [7]	Females= 1470 Males= 745	68.5±11.3	3 years	Gait speed (6m)	Falls	Slower gait speed (0.1m/s decrease), men: OR=1.15 (1.09–1.23), and women: OR=1.05 (1.01–1.10). Slower gait speed predicted falls.
Sanders et al. 2016 [8]	Females= 2179 Males= 1933	74.5±5.8 (≥65)	13 years; 6 years; 21 years	Gait speed (6m)	Falls	Slower gait speed (<0.71m/s): HR=1.3 (1.0-1.5). Slow gait speed predicted falls.

Luukinen et al. 1995 [4]	Females= 640 Males= 376	76.1±4.9 (70-92)	2 years	Gait speed (10m)	Falls	Slower gait speed (<0.77m/s): RR= 2.3 (1.57-3.48). Slow gait speed was an independent risk factor for recurrent falling.
Doi et al. 2013 [9]	Females= 42 Males= 15	79.7±8.2 (≥65)	1 year	Gait speed (10m)	Falls	Slower gait speed (<0.63m/s): OR=0.02 (0.00–0.23). Gait speed predicted risk of falling.
Stenhagen et al. 2013 [10]	Females= 840 Males= 923	(60-93)	3-6 years	Gait speed (15m)	Falls	Slower gait speed: OR=1.77 (1.28–2.46). Slow gait speed predicted falls.
Dargent-Molina et al. 1999 [11]	Females= 5895	80.5±3.8 (≥75)	2 years	Gait speed (6m)	Hip fracture	Slower gait speed: RR=1.8 (1.5–2.0). Gait speed may be a predictor of risk of hip fracture.
Kauppi et al. 2014 [12]	Females= 1331 Males= 969	66.38±8.24 (≥55)	10 years	Gait speed (6.1m)	Hip fracture	Slower gait speed (<0.40m/s): HR=0.70 (0.54–0.92). Gait speed predicted hip fracture.
Wihlborg et al. 2015 [13]	Females= 1044	75.2±0.2	10 years	Gait speed (30m)	Hip fracture	Slower gait speed (<1.3m/s): HR=1.37 (1.14–1.64). Gait speed predicted hip fracture.
Ersoy et al. 2009 [14]	Females= 125	61.4±7.9 (50-79)	½ year	Postural balance (Berg Balance Scale; one-leg stance, eyes open, ≥5s)	Falls	Balance (≤52 points): OR=6.19 (1.32–28.94). Failing one-leg stance (<5s): 0.24 (0.06-1.03). Worse Berg Balance performance predicted falls.
Muir et al. 2010 [15]	Females= 33 Males= 57	79.7±4.8 (60-90)	1 year	Postural balance (Berg Balance Scale)	Falls	Balance impairment: RR=2.00 (1.13–3.56). Impairment balance was a predictor of falls.
Luukinen et al. 1995 [4]	Females= 640 Males= 376	76.1±4.9 (70-92)	2 years	Postural balance (Tinetti Scale)	Falls	Balance (>7 points): RR= 2.9 (1.73-4.92). Impairment balance predicted falls.
Austin et al. 2007 [16]	Females= 1282	±75.2 (70-85)	3 years	Postural balance (tandem stance, eyes open and closed, ≥10s)	Fear of falling	Poor balance, eyes open: OR= 1.78 (1.38–2.28). Poor balance, eyes closed: 1.87 (1.44–2.42). Poor balance predicted developing fear of falling.
Kwan et al. 2012 [6]	Females= 120	74.9±6.4 (65–91)	2 years	Postural balance (near-tandem stance,	Falls	Worse near-tandem stance performance: 0.80 (0.65–0.99). Worse one-leg stance performance: IRR= 0.78 (0.62–0.99).

	Males= 160			eyes open and closed, ≥30s; one-leg stance, eyes open, ≥5s)		Poor balance predicted risk of falls.
Vellas et al. 1997 [17]	Females= 156 Males= 111	72±6.1 (≥60)	3 years	Postural balance (one-leg stance, eyes open, ≥5s)	Falls and injurious falls	Impaired one-leg stance: RR=2.13 (1.04-4.34), for injurious falls. One-leg test predicted injurious falls but not falls.
Mulasso et al. 2017 [18]	Females= 119 Males= 73	73.0±6.2 (≥65)	1 year	Postural balance (one-leg stance, eyes open, 60s)	Falls	One-leg stance: OR=0.99 (0.97–1.01). The one-leg standing balance test was not associated with falls.
Nitz et al. 2013 [19]	Females= 449	59.3±10.6 (40-80)	9 years	Postural balance (bipedal stance, eyes open and closed; one-leg stance, eyes open, 10s. Force platform)	Falls	Unsteady in bipedal stance, eyes closed: OR=1.99 (1.18–3.38), for single falls. Unsteady in bipedal stance, eyes closed: OR=4.21 (1.79–9.92), for multiple falls. Impairment balance detected potential fallers.
Swanenburg et al. 2010 [20]	Females= 225 Males= 45	73±7 (60-90)	1 year	Postural balance (bipedal stance, eyes open and closed, 20s. Force platform)	Falls	Amplitude of in medial–lateral directions during single-task condition: OR=21.8 (3.19-149.25). Impairment balance predicted multiple falls.
Maki et al. 1994 [21]	Females= 83 Males= 17	83±6 (62-96)	1 year	Postural balance (bipedal stance, eyes open and closed, 30s. Force platform)	Falls	Lateral spontaneous-sway amplitude (eyes closed): Mean (SD)=3.35 (2.29), *p*=0.004. Postural balance predicted future risk of falling with moderate accuracy.
Pajala et al. 2008 [22]	Females= 434	(63–76)	1 year	Postural balance (side-by-side, eyes open and closed; tandem stance and semi-tandem stance, eyes open; 30s. Force platform)	Falls	Inability to complete the tandem stance: IRR=4.33 (2.17–8.52). Impairment balance predicted risk of falling.
Frames et al. 2018 [23]	Females= 65 Males= 33	Non obese:	2 years	Postural balance (bipedal stance, eyes	Falls	Obese fallers had higher sway range than non-fallers (*p*=0.001, F=7.44), root mean square values (*p*=0.002, F=6.62) and SD

		77.41±8.49<br>Obese:<br>72.68±7.40		open and closed, 60s. Force platform and an inertial measurement unit affixed at the sternum)		values (*p*=0.002, F=6.62) from the force plate center of pressure time series. Obese fallers had higher sway area than non-fallers (ellipse area, *p*=0.003, F=5.89; and circular area *p*<0.0002, F=8.97), mean velocity (*p*=0.011, F=4.56), mean radius (*p*<0.0001, F=10.47) and mean path length of center of pressure (*p*=0.011, F=4.56). The postural balance test may help detecting fall risk in elderly persons who are obese.
Wihlborg et al. 2015 [13]	Females= 1044	75.2±0.2	10 years	Postural balance (one-leg stance, eyes open and closed, ≥60s)	Hip fracture	Failing the balance test: HR=1.98 (1.18–3.32).<br>Balance predicted hip fracture.
Abu et al. 2018 [5]	Females= 180<br>Males=145	67.7±5.5 (60-89)	½ year	Timed Up&Go (TUG)	Falls	TUG (>8s): IQR: 4.8-17.5, with a sensitivity of 83.95%% (74.1-91.2) and specificity of 32.43% (26.3-39.0).<br>The TUG test had predictive validity for risk of falls.
Asai et al. 2020 [24]	Females= 425<br>Males= 224	±76.2 (≥60)	1 year	TUG	Falls	Worse TUG performance (>7s): OR=1.14 (1.01-1.29), *p*=0.032.<br>Worse TUG predicted risk of falls.
Austin et al. 2007 [16]	Females= 1282	±75.2 (70-85)	3 years	TUG	Fear of falling	TUG (>10s): mean=9.5; IQR=8.1-11.0, for fear of falling, and mean=10.8; IQR=8.7-21.3, for persistent fear.<br>The TUG test predicted fear of falling.
Clemson et al. 2015 [25]	Females= 533<br>Males= 467	±73.4 (65-94)	11 years	TUG	Falls	TUG (>12s): HR=1.02 (1.01-1.04).<br>Worse TUG performance predicted injurious falls.
Doi et al. 2013 [9]	Females= 42<br>Males= 15	79.7±8.2 (≥65)	1 year	TUG	Falls	TUG (>21s): OR=1.08 (1.02–1.15).<br>The TUG test predicted risk of falling.
Ersoy et al. 2009 [14]	Females= 125	61.4±7.9 (50-79)	½ year	TUG	Falls	TUG (>13s): OR= 4.55 1.95-10.61.<br>The TUG test predicted falls.
Kang et al. 2017 [1]	Females= 307<br>Males= 234	67.4±5.6 (60-86)	1 year	TUG	Falls	TUG (>16s): HR=1.07 (1.00–1.14).<br>The TUG test predicted falls.
Kwan et al. 2012 [6]	Females= 120	74.9±6.4 (65–91)	2 years	TUG	Falls	TUG (>11s): IRR=1.27 (1.04–1.56).<br>The TUG test predicted risk of falling.

Mulasso et al. 2017 [18]	Males= 160<br>Females= 119<br>Males= 73	73.0±6.2 (≥65)	1 year	TUG	Falls	TUG (>11s): OR=1.076 (0.94–1.21)<br>The TUG test was not associated with falls.
Nitz et al. 2013 [19]	Females= 449	59.3±10.6 (40-80)	9 years	TUG	Falls	TUG (>8s): 1.32 (1.15, 1.51), for multiple falls.<br>The TUG test predicted multiple falls.
Luukinen et al. 1995 [4]	Females= 640<br>Males= 376	76.1±4.9 (70-92)	2 years	Range Of Motion (ROM) (Hip and knee ROM)	Falls	Hip motion reduced (<120°): RR= 1.4 (0.89-2.04).<br>Knee motion reduced (<100º): 1.4 (0.97-2.04).<br>Reduced ROM was not associated with falls.

HR, Hazard Ratio; IRR, Incidence Rate Ratio; IQR, Interquartile Range; OR, Odss Ratio; ROM, Range Of Motion; RR, Relative Risk; SD, Standard Deviation; TUG, Timed Up&Go test.

Supplementary Table S2. Longitudinal validity of motor fitness and flexibility tests for cognitive outcomes in adults and older adults.

Author	Participants	Sample age, years (Range)	Follow-up	Fitness test	Health outcomes	Main outcomes and conclusions
Ojagbemi et al. 2015 [26*]*	2149 participants (*unspecified sex*)	(≥65)	2 years	Gait speed (3m, 4m)	Cognitive decline	Slower gait speed (>6.52s in 3m; >8.70s in 4m, in total): HR=0.77 (0.43–1.2). Change in gait speed was a significant predictor of reduced cognitive performance.
Hoogendijk et al. 2020 [27]	Females= 2221 Males= 1999	±72.0 (≥55)	9-25 years	Gait speed (4m, 6m, 30m)	Cognitive decline	Slower gait speed (<0.82m/s): HR=1.23 (1.00-1.50), *p*<0.05. Slower gait speed predicted cognitive decline.
Sanders et al. 2016 [8]	Females= 2179 Males= 1933	74.5±5.8 (≥65)	13 years; 6 years; 21 years.	Gait speed (6m)	Persistent cognitive decline	Slower gait speed (<0.71m/s): HR= 2.1 (0.8-5.8). Slow gait speed did not predict persistent cognitive decline.
Stijntjes et al. 2017 [28]	Females= 1647 Males= 1332	(55-90)	5-12 years	Gait speed (6m)	Cognitive decline	No association was found for the group of 55–64 years. Group of 65–74 years: slower gait speed (<0.86m/s) was associated with a steeper decline in global cognitive function (*p*<0.05). Group of 75–85 years: slower gait speed (<0.69m/s) was associated with a steeper decline on each of the cognitive domains (*p*<0.04). Slow gait speed was a marker for cognitive impairment in adults over 65years old.
Tian et al. 2019 [29]	Females= 93 Males= 108	79.3±6.3	5.2 years	Gait speed (6m, 400m)	Cognitive impairment/ Alzheimer's disease	Slower gait speed (<1.02m/s): β=-0.22 (-0.47-0.04), *p*<0.05. Slower gait speed predicted cognitive impairment/Alzheimer's disease.
Buracchio et al. 2010 [30]	Females= 118 Males= 86	79.0±8.8 (≥65)	20 years	Gait speed (9.14m)	Mild cognitive impairment	Gait speed rate of change before converters to mild cognitive impairment= −0.005 m/s/year (−0.010-0.000), *p*=0.07. A decrease of 0.02 m/s/year (*p*=0.001) in gait speed may predict mild cognitive impairment. Changes in gait speed may predict cognitive impairment.

Sakurai et al. 2017 [31]	Females= 106 Males= 117	72.6±4.9 (65–85)	1 year	Gait speed (10m fast gait speed)	Subjective memory complaints	Gait speed (<2.0m/s): OR=0.84 (0.39–1.81). Gait speed did not predict developing subjective memory complaints.
Bullain et al. 2016 [32]	Females= 402 Males= 176	93.3±2.6 (≥90)	2.6 years	Postural balance (one-leg stance, eyes open, 10s)	Dementia	Unable to complete balance test: HR=3.20 (1.91–5.37). Poor balance may predict dementia.
Doi et al. 2019 [33]	Females= 2124 Males= 1962	±72.0 (≥65)	3.6 years	Timed Up&Go (TUG)	Dementia	Worse TUG performance: HR=1.54 (1.01-2.35), *p*=0.04. Worse TUG predicted incidence of dementia.
Donoghue et al. 2017 [34]	Females= 1169 Males= 1081	72.4±6.0 (65–98)	5.9 years	TUG	Cognitive decline	TUG (>10.5s): β=1.00 (0.99-1.00). The TUG test was not significant.

β, standardized regression coefficient; B, non-standardized regression coefficient; SE, standard Error; HR, Hazard Ratio; OR, Odss Ratio; TUG, Timed Up&Go test.

Supplementary Table S3. Longitudinal validity of motor fitness and flexibility tests for depressive symptoms and well-being in adults and older adults.

Author	Participants	Sample age, years (Range)	Follow-up	Fitness test	Health outcomes	Main outcomes and conclusions
Veronese et al. 2017 [35]	Females= 485 Males= 1247	69.3±6.7 (≥50)	2 years	Gait speed (2.5m)	Depressive symptoms	Slower gait speed: OR=1.82 (1.00–3.32). Slow gait speed may predict depression.
Briggs et al. 2019 [36]	3615 participants (*unspecified sex*)	±63.3 (≥50)	2-4 years	Gait speed (4m)	Depressive symptoms	Slower gait speed (<1.3m/s): OR=1.54 (1.08-2.19), p<0.003. Slower gait speed predicted depression.
Veronese et al. 2017[b] [37]	Females= 533 Males= 437	72.5±6.0 (65-96)	4 years	Gait speed (4m)	Depressive symptoms	Slower gait speed (<0.80m/s), women: OR=1.55 (0.90-2.68). Slower gait speed (<0.80m/s), men: OR=2.22 (1.16-4.28). Slow walking speed predicted depression only in men.
Sanders et al. 2012 [38]	Females= 686 Males= 773	68.9±8.5 (≥65)	16 years	Gait speed (6m)	Depressive symptoms	Slower gait speed (<0.55m/s), women: HR=1.22 (0.87–1.72). Slower gait speed (<0.55 m/s), men: HR=1.67 (1.08–2.57). Slow gait speed predicted depressive symptoms only in men.
Davis et al. 2015 [39]	Females= 196 Males= 112	82.5±6.5 (≥70)	1 year	Timed Up&Go (TUG)	Well-being	TUG (>20s): β=-0.30(-0.45, -0.15). The TUG test was a predictor of well-being decrease.

HR, Hazard Ratio; OR, Odss Ratio; β, standardized regression coefficient; TUG, Timed Up&Go test.

Supplementary Table S4. Longitudinal validity of motor fitness and flexibility tests for mobility limitations and disability risk in adults and older adults.

Author	Participants	Sample age, years (Range)	Follow-up	Fitness test	Health outcomes	Main outcomes and conclusions
Makizako et al. 2015 [40]	Females= 463 Males= 485	78.5±3.7 (≥75)	1.25 years	Gait speed (2.4m)	Disability in instrumental activities of daily living (IADL)	Slower gait speed, women (<0.9m/s): OR=0.97 (0.95–0.99). Slower gait speed, men (<1.1m/s): OR=0.98 (0.96–0.99). Gait speed predicted disability in IADL.
Heiland et al. 2018 [41]	Females= 1154 Males= 602	70.6±9.6 (≥60)	9 years	Gait speed (2.4m or 6m)	Disability in IADL	Gait speed limitation (<0.8m/s): HR=3.11 (1.92-5.03). Gait speed predicted disability in IADL.
Abe et al. 2019 [42]	Females= 463 Males= 510	74.6±5.5 (≥65)	4.4 years	Gait speed (5m)	Disability in IADL	Slower gait speed (<1.25m/s): HR=0.86 (0.18-0.92), p<0.001. Slower gait speed predicted disability in IADL.
Makizako et al. 2010 [43]	Females= 146 Males= 119	78±5.6 (68-96)	4 years	Gait speed (5m)	Disability in IADL	Slower gait speed (≥5.6s in total), women: OR=5.31 (1.63-17.22). Slower gait speed, men (≥4.6s in total): OR=1.58 (0.94-2.63). Gait speed predicted disability in IADL only in older women.
Adachi et al. 2019 [44]	Females= 417 Males= 99	±79 (76-82)	2 years	Gait speed (10m)	Disability in IADL	Slower gait speed (<1.13 m/s): AUC=0.80 (0.74-0.86). Slow gait speed predicted disability in IADL.
Laukkanen et al. 2000 [45]	Females= 261 Males= 127	75 and 80	5 years	Gait speed (10m)	Disability in IADL	Slower gait speed: t-value=-0.23, SE=0.06. Reduced gait speed predicted disability in IADL.
Nakamoto et al. 2015 [46]	Females= 466 Males= 495	(40–79)	14 years	Gait speed (10m)	Disability in IADL	Slower gait speed, women (>1 SD increase): OR=0.68 (0.50-0.92). Slower gait speed, men (>1 SD increase): OR= 0.78 (0.59-1.04). Gait speed predicted disability in IADL.
Doi et al. 2020 [47]	Females= 1944 Males= 1752	71.2±4.9 (≥65)	4.1 years	Gait speed (2.4 m)	Mobility disability	Slower gait speed (<1.1m/s): HR=2.06 (1.65-2.57), p<0.001. Slower gait speed predicted mobility disability.
Brach et al. 2012 [48]	Females= 336 Males= 216	79.4±4.1 (≥65)	1 year	Gait speed (4m)	Mobility disability	Slower gait speed (<1m/s): sensitivity, AUC=0.61 (0.47-0.73), specificity, AUC= 0.60 (0.55-0.65). Slow gait may be a predictor of mobility disability.

Deshpande et al. 2013 [49]	622 participants (*unspecified sex*)	(50-85)	3 years	Gait speed (7m)	Mobility disability	Slower gait speed (<1.2m/s): OR= 24.95 (6.03-103.13). Slower gait speed predicted mobility disability.
Rosso et al. 2019 [50]	Females= 171 Males= 166	78.5±2.9 (70-79)	8 years	Gait speed (20m)	Mobility Disability	Slower gait speed (<1.08m/s): HR=0.82 (0.77-0.86). Slower gait speed predicted mobility disability.
Jung et al. 2018 [51]	Females= 746 Males= 602	±76 (≥65)	5.38 years	Gait speed (4m)	Frailty	Slower gait speed (<0.8m/s): β=-0.534, p=0.001. Gait speed may predict frailty status.
Breton et al. 2014 [52]	Females= 652 Males= 613	±73 (68-82)	5 years	Gait speed (4m)	Functional autonomy	Faster gait speed, women (<1.5m/s): R^2=0.37; p<0.001, for greater mobility/autonomy. Faster gait speed, men (<1.7m/s): R^2=0.28; p<0.001, for with greater mobility/autonomy. Gait speed changes could moderately predict functional autonomy, especially in women.
Makizako et al. 2010 [43]	Females= 146 Males= 119	78±5.6 (68-96)	4 years	Postural balance (one-leg stance, eyes open, 60s)	Disability in IADL	Balance, women: OR=0.98 (0.95-1.02). Balance, men: OR=0.98 (0.95-1.00). Balance did not predict disability in IADL.
Nakamoto et al. 2015 [46]	Females= 466 Males= 495	(40–79)	14 years	Postural balance (bipedal stance, eyes open, 60s. Force platform)	Disability in IADL	A 1-SD increase in sway area with eyes open, women: OR=1.49 (1.17–1.90). A 1-SD increase in sway area with eyes open, men: OR= 0.92 (0.71–1.20). Sway area increases might predict disability in IADL, only in women.
Breton et al. 2014 [52]	Females= 652 Males= 613	±73 (68-82)	5 years	Postural balance (one-leg stance, eyes open, 60s)	Functional autonomy	Balance, women: R^2=0.37; p<0.001. Balance, men: R^2=0.28; p<0.001. Balance could moderately predict functional autonomy, especially in women.
Savva et al. 2013 [53]	Females= 925 Males= 889	±70 (≥65)	4 years	TUG	Frailty	TUG (>16s): AUC=0.87 for frailty. TUG (>12s): AUC=0.73 for prefrailty. The TUG test identified prefrailty and frailty.

Breton et al. 2014 [52]	Females= 652 Males= 613	±73 (68-82)	5 years	TUG	Functional autonomy	TUG (>11s), women: β (SE)= 0.56 (0.13); *p*<0.001. TUG (>10.5s), men: β (SE)= 0.42 (0.12); *p*<0.001. The TUG test predicted decline in functional autonomy.
Ward et al. 2016 [54]	Females= 261 Males= 130	76.5±7.1 (≥65)	1-2 years	Range Of Motion (ROM) (knee flexion-extension, ankle)	Mobility disability	Restricted knee flexion: OR=2.03 (1.24-3.35). ROM predicted mobility disability.
Ward et al. 2016 [54]	Females= 261 Males= 130	76.5±7.1 (≥65)	1-2 years	Speed of movement (leg velocity, reaction time, rapid leg coordination)	Mobility disability	Slower leg velocity: OR=2.35 (1.21-4.58). Leg velocity predicted mobility disability.
Adachi et al. 2019 [44]	Females= 417 Males= 99	±79 (76-82)	2 years	Maximum step length	Disability in IADL	Maximum step length: AUC=0.81 (0.75-0.87). Maximum step length was a predictor for declined walking speed and predicted disability in IADL.
Schroll et al. 1997 [55]	Females= 146 Males= 113	(75-80)	5 years	Stair mounting (40-50cm step)	Disability in IADL	Disability to complete the stair mounting test, women (40 cm): RR=4.14, *p*=0.09. Disability to complete the stair mounting test, men (50 cm): RR=4.07, *p*<0.001. Inability to mount a 50cm step was a predictor of disability in IADL in men.

β, standardized regression coefficient; IADL, Disability in instrumental activities of daily living; AUC, Area Under the Curve; HR, Hazard Ratio; OR, Odss Ratio; ROM, Range Of Motion; RR, Relative Risk; SD, Standard Deviation; SE, Standard Error; TUG, Timed Up&Go test.

Supplementary Table S5. Longitudinal validity of motor fitness and flexibility tests for CVD risk and mortality in adults and older adults.

Author	Participants	Sample age, years (Range)	Follow-up	Fitness test	Health outcomes	Main outcomes and conclusions
Heiland et al. 2018 [41]	Females= 1154<br>Males= 602	70.6±9.6 (≥60)	9 years	Gait speed (2.4m or 6m)	CVD risk	Slower gait speed (<0.8m/s): HR=1.09 (1.02–1.17), for group <78 years.<br>Slower gait speed (<0.8m/s): HR= 0.98 (0.92–1.03), for group ≥78 years.<br>Slow gait speed predicted CVD risk only in adults <78 years.
Elbaz et al. 2013 [56]	Females= 1817<br>Males= 4450	(35-55)	6.4 years	Gait speed (2.4m)	All-cause mortality	Slower gait speed, women (<1.09m/s), men (<1.26m/s): HR= 2.19 (1.54–3.11).<br>Gait speed was a marker of mortality risk.
Zucchelli et al. 2019 [57]	Females= 2182<br>Males= 1181	74.7±11.2 (≥60)	3-5 years	Gait speed (2.4m, 6m)	All-cause mortality and unplanned hospitalization	Slower gait speed (<0.8m/s): AUC=0.85 (0.83-0.87), for 3-years mortality; AUC=0.85 (0.83-0.86), for 5-years mortality.<br>Slower gait speed (<0.8m/s): AUC=0.70 (0.68-0.72) for 3-years hospitalization.<br>Slower gait speed predicted mortality and unplanned hospitalization.
Andrasfay et al. 2020 [58]	Females= 430<br>Males= 457	70.1±8.7 (≥60)	4 years	Gait speed (3m)	All-cause mortality	A 1-SD increase in gait speed (<0.1m/s): HR=0.70 (0.52-0.94), $p<0.05$.<br>Slower gait speed predicted mortality risk.
Camargo et al. 2016 [59]	Females= 1175<br>Males= 1001	62±9 (35-84)	11 years	Gait speed (4m)	Stroke	Slower gait speed (<1.0m/s): HR=1.13 (0.88–1.45).<br>Slow gait speed was not significantly associated with risk of stroke.
Jung et al. 2018 [51]	Females= 746<br>Males= 602	±76 (≥65)	5.38 years	Gait speed (4m)	All-cause mortality	Gait speed (<0.8m/s): HR=3.37 (1.25–9.08).<br>Gait speed may predict mortality.
Niiranen et al. 2019 [60]	Females= 1852<br>Males= 1601	54.7±9.2 (45-74)	8 years	Gait speed (4m)	All-cause mortality and Cardiovascular Disease (CVD) risk	Slower gait speed (<1.29m/s): women, HR=3.72 (2.53-5.47), $p<0.001$; men, HR=2.57 (1.77-3.74), $p<0.001$, for mortality.<br>Slower gait speed (<0.99m/s): HR=2.95 (2.26-3.85), $p<0.05$, for CVD risk.

						Slower gait speed predicted incidence of CVD risk and mortality.
Hoogendijk et al. 2020 [27]	Females= 2221 Males= 1999	±72.0 (≥55)	9-25 years	Gait speed (4m, 6m, 30m)	All-cause mortality	Slower gait speed (<0.82m/s): HR=1.70 (1.30-6.28), *p*<0.05. Slower gait speed predicted mortality.
Abe et al. 2019 [42]	Females= 463 Males= 510	74.6±5.5 (≥65)	4.5 years	Gait speed (5m)	All-cause mortality	Slower gait speed (<1.25m/s): HR=0.93 (0.88-1.00), *p*=0.038. Slower gait speed mortality risk.
Nofuji et al. 2016 [61]	1085 participants (*unspecified sex*)	(65-89)	10.5 years	Gait speed (5m)	All-cause mortality and cause-specific mortality ((CVD), cancer, other-causes and unknown causes))	Slower gait speed, women (<0.94m/s), men (<1.10m/s): HR=1.95 (1.39-2.73), all-cause death. Slower gait speed, women (<0.94m/s), men (<1.10m/s): HR=2.80 (1.55-5.05), CVD death. Slower gait speed women (<0.94m/s), men (<1.10m/s): HR=2.08 (1.14-3.77), other-cause death. Slow gait speed predicted all-cause, CVD, and other-cause mortality, but not cancer mortality.
Lee et al. 2017 [62]	Females= 407 Males= 504	65.3±9.3	4.1 years	Gait speed (3m)	All-cause mortality and CVD risk	Slower gait speed (<0.8m/s): HR=1.3 (0.6–2.5), for mortality. Slower gait speed (<0.8m/s): HR=0.8 (0.2–3.3), for CVD risk. Not associations were found between gait speed and any studied variable.
Blain et al. 2010 [63]	Females= 1548	±79 (77-81)	8 years	Gait speed (6m)	All-cause mortality	Slower gait speed (<0.8m/s): HR=1.33 (0.94-1.88). Slow gait speed predicted mortality.
Sabia et al. 2014 [64]	4016 participants (*unspecified sex*)	73.4±4.7 (65–85)	12 years	Gait speed (6m)	All-cause mortality	Slower gait speed decline/year (<−0.08m/s): HR=1.40 (1.02-1.92). Fast gait speed decline predicted mortality.
Sanders et al. 2016 [8]	Females= 2179 Males= 1933	74.5±5.8 (≥65)	13 years; 6 years; 21 years	Gait speed (6m)	All-cause mortality	Slower gait speed (<0.71m/s): HR=2.1 (1.6-2.6 45). Slow gait speed predicted mortality.
Georgiopoulou et al. 2016 [65]	Females= 1529 Males= 1406	73.6±2.9 (70-79)	10 years	Gait speed (20m)	All-cause mortality and CVD risk	Slower gait speed (<1.35m/s): HR=0.34 (0.24-0.48), mortality. Slower gait speed (<1.35m/s): HR=0.36 (0.22-0.60), CVD. Slow gait speed test may predict mortality and CVD risk.

Idland et al. 2013 [66]	Females= 113	±79.5 (75–92)	9 years	Gait speed (29m)	All-cause mortality	Slower gait speed (<1.1m/s): OR=0.05 (0.02-0.16). Slow gait speed predicted mortality risk.
Looker 2015 [67]	Females= 1475 Males= 1500	(≥50)	3 years	Gait speed (6.15m)	Mortality risk by dysmobility	Slower gait speed (<1.0m/s): HR=3.63 (2.69-4.90). Dysmobility (by slow gait speed) was associated with increased mortality risk.
Blain et al. 2010 [63]	Females= 1548	±79 (77-81)	8 years	Postural balance (side-by-side, tandem stance, to complete 10-foot taps, eyes open, s)	All-cause mortality	Balance (≥4.6s): HR= 1.67 (1.21-2.31). Poor balance predicted mortality.
Cooper et al. 2014 [68]	Females= 1411 Males= 1355	53	13 years	Postural balance (one-leg stance, eyes closed, 30s)	All-cause mortality	Unable to perform standing balance test: HR=3.40 (2.05-5.63). Inability to perform balance test was a strongly predictor of mortality.
Nofuji et al. 2016 [61]	1085 participants (*unspecified sex*)	(65-89)	10.5 years	Postural balance (one-leg stance, eyes open, 60s)	All-cause mortality and cause-specific mortality (CVD, cancer, other-causes and unknown causes)	One-leg stance (<18 score): HR= 1.91 (1.39-2.63), all-cause death. One-leg stance (<18 score): HR= 2.67 (1.54-4.62), CVD death. One-leg stance (<18 score): HR= 2.60 (1.45-4.69), other-causes death. Poor balance predicted all-cause, CVD, and other-cause mortality, but not cancer mortality.
Idland et al. 2013 [66]	Females= 113	±79.5 (75–92)	9 years	Dynamic balance (Functional reach test, cm)	All-cause mortality	Poorer functional reach: OR=0.92 (0.88-0.95). Poorer functional reach predicted mortality risk.
Bravell et al. 2017 [69]	Females= 343 Males= 242	66±9 (60-91)	19 years	Flexibility (Touch-toes test)	All-cause mortality	Touch-toes test: HR=0.99 (0.42-2.31). The predictive validity of this flexibility test was not significant.

CVD, Cardiovascular Disease; HR, Hazard Ratio; OR, Odss Ratio.

REFERENCES

1. Kang L, Han PP, Wang JZ, Ma YX, Jia LY, Fu LY, et al. Timed Up and Go Test can predict recurrent falls: a longitudinal study of the community-dwelling elderly in China. Clinical Interventions in Aging. 2017;12:2009-16.
2. Quach L, Galica AM, Jones RN, Procter-Gray E, Manor B, Hannan MT, et al. The Nonlinear Relationship Between Gait Speed and Falls: The Maintenance of Balance, Independent Living, Intellect, and Zest in the Elderly of Boston Study. Journal of the American Geriatrics Society. 2011;59(6):1069-73.
3. Callisaya ML, Ayers E, Barzilai N, Ferrucci L, Guralnik JM, Lipton RB, et al. Motoric Cognitive Risk Syndrome and Falls Risk: A Multi-Center Study. Journal of Alzheimers Disease. 2016;53(3):1043-52.
4. Luukinen H, Koski K, Laippala P, Kivela SL. PREDICTORS FOR RECURRENT FALLS AMONG THE HOME-DWELLING ELDERLY. Scandinavian Journal of Primary Health Care. 1995;13(4):294-9.
5. Abu Samah Z, Singh DKA, Murukesu RR, Shahar S, Nordin NAM, Omar MA, et al. Discriminative and Predictive Ability of Physical Performance Measures in Identifying Fall Risk among Older Adults. Sains Malaysiana. 2018;47(11):2769-76.
6. Kwan MM, Lin SI, Close JC, Lord SR. Depressive symptoms in addition to visual impairment, reduced strength and poor balance predict falls in older Taiwanese people. Age and ageing. 2012;41(5):606-12.
7. Muraki S, Akune T, Oka H, Ishimoto Y, Nagata K, Yoshida M, et al. Physical performance, bone and joint diseases, and incidence of falls in Japanese men and women: a longitudinal cohort study. Osteoporosis International. 2013;24(2):459-66.
8. Sanders JB, Bremmer MA, Comijs HC, van de Ven PM, Deeg DJH, Beekman ATF. Gait Speed and Processing Speed as Clinical Markers for Geriatric Health Outcomes. American Journal of Geriatric Psychiatry. 2016;25:374-85.
9. Doi T, Hirata S, Ono R, Tsutsumimoto K, Misu S, Ando H. The harmonic ratio of trunk acceleration predicts falling among older people: results of a 1-year prospective study. Journal of Neuroengineering and Rehabilitation. 2013;10.
10. Stenhagen M, Ekstrom H, Nordell E, Elmstahl S. Falls in the general elderly population: a 3- and 6-year prospective study of risk factors using data from the longitudinal population study 'Good ageing in Skane'. Bmc Geriatrics. 2013;13.
11. Dargent-Molina P, Schott AM, Hans D, Favier F, Grandjean H, Baudoin C, et al. Separate and combined value of bone mass and gait speed measurements in screening for hip fracture risk: Results from the EPIDOS study. Osteoporosis International. 1999;9(2):188-92.

12. Kauppi M, Stenholm S, Impivaara O, Maki J, Heliovaara M, Jula A. Fall-related risk factors and heel quantitative ultrasound in the assessment of hip fracture risk: a 10-year follow-up of a nationally representative adult population sample. Osteoporosis International. 2014;25(6):1685-95.
13. Wihlborg A, Englund M, Akesson K, Gerdhem P. Fracture predictive ability of physical performance tests and history of falls in elderly women: a 10-year prospective study. Osteoporosis International. 2015;26(8):2101-9.
14. Ersoy Y, MacWalter RS, Durmus B, Altay ZE, Baysal O. Predictive Effects of Different Clinical Balance Measures and the Fear of Falling on Falls in Postmenopausal Women Aged 50 Years and Over. Gerontology. 2009;55(6):660-5.
15. Muir SW, Berg K, Chesworth BM, Klar N, Speechley M. Modifiable Risk Factors Identify People Who Transition from Non-fallers to Fallers in Community-Dwelling Older Adults: A Prospective Study. Physiotherapy Canada. 2010;62(4):358-67.
16. Austin N, Devine A, Dick I, Prince R, Bruce D. Fear of falling in older women: a longitudinal study of incidence, persistence, and predictors. Journal of the American Geriatrics Society. 2007;55(10):1598-603.
17. Vellas BJ, Wayne SJ, Romero L, Baumgartner RN, Rubenstein LZ, Garry PJ. One-leg balance is an important predictor of injurious falls in older persons. Journal of the American Geriatrics Society. 1997;45:735-8.
18. Mulasso A, Roppolo M, Gobbens RJ, Rabaglietti E. Mobility, balance and frailty in community-dwelling older adults: at is the best 1-year predictor of falls? Geriatrics & Gerontology International. 2017;17(10):1463-9.
19. Nitz JC, Stock L, Khan A. Health-related predictors of falls and fractures in women over 40. Osteoporosis international : a journal established as result of cooperation between the European Foundation for Osteoporosis and the National Osteoporosis Foundation of the USA. 2013;24:613-21.
20. Swanenburg J, de Bruin ED, Uebelhart D, Mulder T. Falls prediction in elderly people: A 1-year prospective study. Gait & Posture. 2010;31(3):317-21.
21. Maki BE, Holliday PJ, Topper AK. A PROSPECTIVE-STUDY OF POSTURAL BALANCE AND RISK OF FALLING IN AN AMBULATORY AND INDEPENDENT ELDERLY POPULATION. Journals of Gerontology. 1994;49(2):M72-M84.
22. Pajala S, Era P, Koskenvuo M, Kaprio J, Tormakangas T, Rantanen T. Force platform balance measures as predictors of indoor and outdoor falls in community-dwelling women aged 63-76 years. Journals of Gerontology Series a-Biological Sciences and Medical Sciences. 2008;63(2):171-8.

23. Frames CW, Soangra R, Lockhart TE, Lach J, Ha DS, Roberto KA, et al. Dynamical Properties of Postural Control in Obese Community-Dwelling Older Adults. Sensors. 2018;18(6).
24. Asai T, Oshima K, Fukumoto Y, Yonezawa Y, Matsuo A, Misu S. Does dual-tasking provide additional value in timed "up and go" test for predicting the occurrence of falls? A longitudinal observation study by age group (young-older or old-older adults). Aging Clinical and Experimental Research. 2020.
25. Clemson L, Kendig H, Mackenzie L, Browning C. Predictors of Injurious Falls and Fear of Falling Differ: An 11-Year Longitudinal Study of Incident Events in Older People. Journal of Aging and Health. 2015;27(2):239-56.
26. Ojagbemi A, D'Este C, Verdes E, Chatterji S, Gureje O. Gait speed and cognitive decline over 2 years in the Ibadan study of aging. Gait & Posture. 2015;41(2):736-40.
27. Hoogendijk EO, Rijnhart JJM, Skoog J, Robitaille A, van den Hout A, Ferrucci L, et al. Gait speed as predictor of transition into cognitive impairment: Findings from three longitudinal studies on aging. Experimental Gerontology. 2020;129.
28. Stijntjes M, Aartsen MJ, Taekema DG, Gussekloo J, Huisman M, Meskers CGM, et al. Temporal Relationship Between Cognitive and Physical Performance in Middle-Aged to Oldest Old People. Journals of Gerontology Series a-Biological Sciences and Medical Sciences. 2017;72(5):662-8.
29. Tian Q, Resnick SM, Studenski SA, Ferrucci L. Lap Time Variability From a 400-m Walk Is Associated With Future Mild Cognitive Impairment and Alzheimer's Disease. Journal of the American Medical Directors Association. 2019;20(12):1535-+.
30. Buracchio T, Dodge HH, Howieson D, Wasserman D, Kaye J. The Trajectory of Gait Speed Preceding Mild Cognitive Impairment. Archives of Neurology. 2010;67(8):980-6.
31. Sakurai R, Suzuki H, Ogawa S, Kawai H, Yoshida H, Hirano H, et al. Fear of falling, but not gait impairment, predicts subjective memory complaints in cognitively intact older adults. Geriatrics & Gerontology International. 2017;17(7):1125-31.
32. Bullain SS, Corrada MM, Perry SM, Kawas CH. Sound Body Sound Mind? Physical Performance and the Risk of Dementia in the Oldest-Old: The 90+Study. Journal of the American Geriatrics Society. 2016;64(7):1408-15.
33. Doi T, Tsutsumimoto K, Nakakubo S, Kim MJ, Kurita S, Hotta R, et al. Physical Performance Predictors for Incident Dementia Among Japanese Community-Dwelling Older Adults. Physical Therapy. 2019;99(9):1132-40.

34. Donoghue O, Feeney J, O'Leary N, Kenny RA. Baseline Mobility is Not Associated with Decline in Cognitive Function in Healthy Community-Dwelling Older Adults: Findings From The Irish Longitudinal Study on Ageing (TILDA). American Journal of Geriatric Psychiatry. 2017;26:438-48.
35. Veronese N, Solmi M, Maggi S, Noale M, Sergi G, Manzato E, et al. Frailty and incident depression in community-dwelling older people: results from the ELSA study. International Journal of Geriatric Psychiatry. 2017;32(12):E141-E9.
36. Briggs R, Carey D, Claffey P, McNicholas T, Donoghue O, Kennelly SP, et al. Do Differences in Spatiotemporal Gait Parameters Predict the Risk of Developing Depression in Later Life? Journal of the American Geriatrics Society. 2019;67(5):1050-6.
37. Veronese N, Stubbs B, Trevisan C, Bolzetta F, De Rui M, Solmi M, et al. Poor Physical Performance Predicts Future Onset of Depression in Elderly People: Progetto Veneto Anziani Longitudinal Study. Physical Therapy. 2017;97(6):659-68.
38. Sanders JB, Bremmer MA, Deeg DJH, Beekman ATF. Do Depressive Symptoms and Gait Speed Impairment Predict Each Other's Incidence? A 16-Year Prospective Study in the Community. Journal of the American Geriatrics Society. 2012;60(9):1673-80.
39. Davis JC, Best JR, Bryan S, Li LC, Hsu CL, Gomez C, et al. Mobility Is a Key Predictor of Change in Well-Being Among Older Adults Who Experience Falls: Evidence From the Vancouver Falls Prevention Clinic Cohort. Archives of physical medicine and rehabilitation. 2015;96(9):1634-40.
40. Makizako H, Shimada H, Doi T, Tsutsumimoto K, Lee S, Hotta R, et al. Cognitive Functioning and Walking Speed in Older Adults as Predictors of Limitations in Self-Reported Instrumental Activity of Daily Living: Prospective Findings from the Obu Study of Health Promotion for the Elderly. International Journal of Environmental Research and Public Health. 2015;12(3):3002-13.
41. Heiland EG, Welmer AK, Wang R, Santoni G, Fratiglioni L, Qiu CX. Cardiovascular Risk Factors and the Risk of Disability in Older Adults: Variation by Age and Functional Status. Journal of the American Medical Directors Association. 2018;20:208-+.
42. Abe T, Kitamura A, Taniguchi Y, Amano H, Seino S, Yokoyama Y, et al. Pathway from gait speed to incidence of disability and mortality in older adults: A mediating role of physical activity. Maturitas. 2019;123:32-6.
43. Makizako H, Furuna T, Yoshida H, Shimada H, Satoh K, Akanuma T, et al. Usual Walking Speed Predicts Decline of Functional Capacity among Community-Dwelling Older Japanese Women: a 4-year Longitudinal Study. Journal of Physical Therapy Science. 2010;22(4):405-12.

44. Adachi T, Kamiya K, Kono Y, Iwatsu K, Shimizu Y, Honda I, et al. Estimation of reduced walking speed using simple measurements of physical and psychophysiological function in community-dwelling elderly people: a cross-sectional and longitudinal study. Aging Clinical and Experimental Research. 2019;31(1):59-66.
45. Laukkanen P, Leskinen E, Kauppinen M, Sakari-Rantala R, Heikkinen E. Health and functional capacity as predictors of community dwelling among elderly people. Journal of Clinical Epidemiology. 2000;53(3):257-65.
46. Nakamoto M, Otsuka R, Yuki A, Nishita Y, Tange C, Tomida M, et al. Higher gait speed and smaller sway area decrease the risk for decline in higher-level functional capacity among middle-aged and elderly women. Archives of Gerontology and Geriatrics. 2015;61(3):429-36.
47. Doi T, Nakakubo S, Tsutsumimoto K, Kim MJ, Kurita S, Ishii H, et al. Spatio-temporal gait variables predicted incident disability. Journal of Neuroengineering and Rehabilitation. 2020;17(1).
48. Brach JS, Wert D, VanSwearingen JM, Newman AB, Studenski SA. Use of Stance Time Variability for Predicting Mobility Disability in Community-Dwelling Older Persons: A Prospective Study. Journal of Geriatric Physical Therapy. 2012;35(3):112-7.
49. Deshpande N, Metter EJ, Guralnik J, Bandinelli S, Ferrucci L. Predicting 3-Year Incident Mobility Disability in Middle-Aged and Older Adults Using Physical Performance Tests. Archives of Physical Medicine and Rehabilitation. 2013;94(5):994-7.
50. Rosso AL, Metti AL, Faulkner K, Brach JS, Studenski SA, Redfern M, et al. Associations of Usual Pace and Complex Task Gait Speeds With Incident Mobility Disability. Journal of the American Geriatrics Society. 2019;67(10):2072-6.
51. Jung HW, Jang IY, Lee CK, Yu SS, Hwang JK, Jeon C, et al. Usual gait speed is associated with frailty status, institutionalization, and mortality in community-dwelling rural older adults: a longitudinal analysis of the Aging Study of Pyeongchang Rural Area. Clinical Interventions in Aging. 2018;13:1079-89.
52. Breton E, Beloin F, Fortin C, Martin A, Ouellet ME, Payette H, et al. Gender-specific associations between functional autonomy and physical capacities in independent older adults: Results from the NuAge study. Archives of Gerontology and Geriatrics. 2014;58(1):56-62.
53. Savva GM, Donoghue OA, Horgan F, O'Regan C, Cronin H, Kenny RA. Using Timed Up-and-Go to Identify Frail Members of the Older Population. Journals of Gerontology Series a-Biological Sciences and Medical Sciences. 2013;68(4):441-6.
54. Ward RE, Beauchamp MK, Latham NK, Leveille SG, Percac-Lima S, Kurlinski L, et al. Neuromuscular Impairments Contributing to Persistently Poor and Declining Lower-Extremity Mobility Among Older Adults: New Findings Informing Geriatric Rehabilitation. Archives of Physical Medicine and Rehabilitation. 2016;97(8):1316-22.

55. Schroll M, Avlund K, Davidsen M. Predictors of five-year functional ability in a longitudinal survey of men and women aged 75 to 80. The 1914-population in Glostrup, Denmark. Aging-Clinical and Experimental Research. 1997;9(1-2):143-52.
56. Elbaz A, Sabia S, Brunner E, Shipley M, Marmot M, Kivimaki M, et al. Association of walking speed in late midlife with mortality: results from the Whitehall II cohort study. Age. 2013;35(3):943-52.
57. Zucchelli A, Vetrano DL, Grande G, Calderon-Larranaga A, Fratiglioni L, Marengoni A, et al. Comparing the prognostic value of geriatric health indicators: a population-based study. Bmc Medicine. 2019;17(1).
58. Andrasfay T. Changes in Physical Functioning as Short-Term Predictors of Mortality. Journals of Gerontology Series B-Psychological Sciences and Social Sciences. 2020;75(3):630-9.
59. Camargo EC, Weinstein G, Beiser AS, Tan ZS, DeCarli C, Kelly-Hayes M, et al. Association of Physical Function with Clinical and Subclinical Brain Disease: The Framingham Offspring Study. Journal of Alzheimers Disease. 2016;53(4):1597-608.
60. Niiranen TJ, Enserro DM, Larson MG, Vasan RS. Multisystem Trajectories Over the Adult Life Course and Relations to Cardiovascular Disease and Death. Journals of Gerontology Series a-Biological Sciences and Medical Sciences. 2019;74(11):1778-85.
61. Nofuji Y, Shinkai S, Taniguchi Y, Amano H, Nishi M, Murayama H, et al. Associations of Walking Speed, Grip Strength, and Standing Balance With Total and Cause-Specific Mortality in a General Population of Japanese Elders. Journal of the American Medical Directors Association. 2016;17(2).
62. Lee WJ, Peng LN, Chiou ST, Chen LK. Physical Health Indicators Improve Prediction of Cardiovascular and All-cause Mortality among Middle-Aged and Older People: a National Population-based Study. Scientific Reports. 2017;7.
63. Blain H, Carriere I, Sourial N, Berard C, Favier F, Colvez A, et al. Balance and walking speed predict subsequent 8-year mortality independently of current and intermediate events in well-functioning women aged 75 years and older. Journal of Nutrition Health & Aging. 2010;14(7):595-600.
64. Sabia S, Dumurgier J, Tavernier B, Head J, Tzourio C, Elbaz A. Change in Fast Walking Speed Preceding Death: Results From a Prospective Longitudinal Cohort Study. Journals of Gerontology Series a-Biological Sciences and Medical Sciences. 2014;69(3):354-62.
65. Georgiopoulou VV, Kalogeropoulos AP, Chowdhury R, Binongo JNG, Bibbins-Domingo K, Rodondi N, et al. Exercise Capacity, Heart Failure Risk, and Mortality in Older Adults: The Health ABC Study. American Journal of Preventive Medicine. 2016;52:144-53.

66. Idland G, Pettersen R, Avlund K, Bergland A. Physical performance as long-term predictor of onset of activities of daily living (ADL) disability: A 9-year longitudinal study among community-dwelling older women. Archives of Gerontology and Geriatrics. 2013;56(3):501-6.

67. Looker AC. Dysmobility syndrome and mortality risk in US men and women age 50 years and older. Osteoporosis International. 2015;26(1):93-102.

68. Cooper R, Strand BH, Hardy R, Patel KV, Kuh D. Physical capability in mid-life and survival over 13 years of follow-up: British birth cohort study. BMJ (Clinical research ed). 2014;348:g2219.

69. Bravell ME, Finkel D, Aslan AD, Reynolds CA, Hallgren J, Pedersen NL. Motor functioning differentially predicts mortality in men and women. Archives of Gerontology and Geriatrics. 2017;72:6-11.
